# Contrasting Assembly and Network Roles of Abundant and Rare Bacteria in Reservoir and Soil Habitats

**DOI:** 10.3390/biology14091291

**Published:** 2025-09-18

**Authors:** Cuixia Zhang, Haiming Li, Mengdi Li, Sihui Su, Han Xiao, Xiaodong Zhang, Qian Zhang

**Affiliations:** 1 College of Marine and Environmental Sciences, Tianjin University of Science and Technology, Tianjin 300457, China; zhangcuixia@tust.edu.cn (C.Z.);; 2 Key Laboratory of Marine Resource Chemistry and Food Technology, Ministry of Education, Tianjin 300457, China; 3 Tianjin Key Laboratory of Marine Resources and Chemistry, Tianjin 300457, China; 4 Binhai Laboratory of Groundwater Utilization and Protection, Tianjin University of Science and Technology, Tianjin 300457, China; 5 Chinese Research Academy of Environmental Sciences, Beijing 100012, China

**Keywords:** bacterial community, rare taxa, abundant taxa, diversity, community assembly, co-occurrence networks, reservoir, soil, saline wetland

## Abstract

Reservoir water and the adjacent soil are ecologically interconnected yet distinct microhabitats in saline coastal wetland ecosystems, but direct comparisons of their bacterial community composition and assembly remain limited. Here, we integrated high-throughput 16S rRNA gene sequencing with statistical, null model, and network analyses to compare diversity patterns, assembly mechanisms, and interactions of abundant and rare bacterial taxa in both habitats. Soil communities exhibited greater taxonomic diversity but a lower overall abundance, while reservoir communities displayed a pronounced vertical stratification, in contrast to the more spatially uniform soil communities at the sampled scale. Key environmental drivers differed: salinity (reflecting the harsh saline context) and nutrient levels structured reservoir communities, whereas the nutrient availability and cation exchange capacity predominated in soils. Stochastic processes mainly governed the assembly of abundant taxa in both habitats, whereas deterministic selection more strongly structured rare taxa, especially in soils subject to harsh saline conditions. The co-occurrence network analysis revealed higher connectivity and modularity in soils, with moderate taxa acting as critical connectors between modules. In contrast, rare taxa played a pivotal role in sustaining network stability in the reservoir. Together, these findings demonstrate distinct, habitat-dependent assembly mechanisms and ecological roles of abundant and rare bacterial taxa in saline coastal wetland microhabitats, providing insights that can inform wetland conservation and ecosystem management.

## Simple Summary

Wetlands are rich and varied environments where water and soil exist side by side, each hosting different communities of tiny living things called bacteria. However, we still know little about how these bacterial groups differ between reservoirs and nearby soils. In this study, we compared the types and relationships of common and rare bacteria found in reservoir water and the soils around it. We discovered that soil has more types of bacteria, though in lower overall numbers, while reservoirs contain fewer types but in higher total amounts. The bacteria in reservoirs and soil respond to different environmental factors; in reservoirs, nutrients and salinity (salt levels) are the most important, while soil bacteria are shaped by nutrients and the soil’s properties under saline conditions. We also found that common bacteria are mostly affected by random events, but rare bacteria, especially in soil, are more influenced by the environment. When examining how bacteria interact, we found that soil communities are more tightly connected, while in reservoirs, rare bacteria are important for keeping the system stable. Understanding these differences helps us protect and manage wetlands more effectively.

## 1. Introduction

Coastal wetlands are dynamic and complex ecosystems shaped by multiple drivers, including the freshwater inflow from rivers, surface runoff, and groundwater discharge, as well as evaporation, precipitation, and tidal intrusion [1]. The freshwater input delivers nutrients and sediments and helps maintain salinity gradients that govern biota and key ecosystem processes. Within these systems, reservoirs—engineered water bodies designed to alleviate freshwater shortages [2]—often form distinct habitats, especially when they are hydrologically connected and ecologically interactive with the surrounding natural wetlands. While differing in origin from natural lakes and wetlands, reservoirs also function as important aquatic habitats, contributing to the overall biodiversity and ecosystem functioning of wetland complexes.

Water bodies and soils represent two typical yet distinct microhabitats that are interconnected via the hydrological exchange within wetland systems. Wetland soils are influenced by multiple environmental factors, with reservoirs playing a critical role in regulating adjacent soil conditions. Numerous studies have highlighted the hydrological connectivity between reservoirs and the surrounding groundwater in coastal regions [3,4]. Reservoirs affect groundwater levels through lateral seepage and vertical water exchange [5], leading to fluctuations that include marked seasonal variations in the water table depth associated with wet/dry periods and reservoir operations. These variations periodically expand or contract the capillary fringe, thereby modulating the adjacent soil’s moisture and solute dynamics (e.g., salt and nutrients) via capillary rise and plant-mediated processes. When groundwater tables are shallow, the capillary rise can extend into the near-surface soil, but its effective depth varies with soil hydraulic properties (e.g., texture, structure, organic matter) and with soil heterogeneity (e.g., interbedded sandy and clayey layers, organic-rich lenses) that create spatial contrasts in hydraulic conductivity and, consequently, in the height and rate of the capillary rise [6]. Concurrently, rainfall, surface runoff, and plant roots transport materials between the upper soil and the reservoir. Evapotranspiration further modulates this balance by imposing a strong upward water demand—particularly in warm, dry seasons—that can draw down near-surface moisture, limit the upward capillary supply to the root zone, and effectively truncate the capillary fringe [7]. Under appropriate site-specific hydrogeological conditions (e.g., shallow water tables and permeable strata), these bidirectional hydrological pathways can closely couple the reservoir and adjacent soils, co-regulating the moisture and solute dynamics and shaping microbial communities.

Bacterial communities are key constituents of these microhabitats, facilitating carbon cycling and driving vital ecological and geochemical processes across terrestrial and aquatic environments [8,9]. Numerous studies show that abundant and rare bacterial taxa play critical roles in ecosystem functioning but respond differently to environmental variation [10,11,12,13,14]. Of particular relevance here, the “rare biosphere” refers to microbial taxa that occur at low relative abundances within a given habitat. Within these communities, dormant propagules collectively form a microbial “seed bank”, which can enhance community resilience through their diversity and potential for resuscitation under favorable conditions [15,16]. Importantly, rare taxa may include both dormant members and metabolically active low-abundance populations. Recent research highlights the ecological importance of the rare biosphere, as rare species frequently demonstrate high metabolic activity and genetic diversity, playing essential roles in ecosystem stability and function [17,18]. Importantly, rarity is context-dependent: a taxon may be rare in one habitat but abundant in another [19,20], which is a central focus of our cross-habitat analysis of the reservoir and adjacent soils. Clarifying the ecological strategies of abundant and rare taxa for adapting to different habitats is critical for developing effective conservation approaches and sustaining wetland ecosystem services.

Community assembly is shaped by both deterministic processes (e.g., environmental filtering and biotic interactions) and stochastic processes (e.g., ecological drift, dispersal, and historical contingency) [21,22]. These processes are not mutually exclusive; they operate simultaneously, and their relative importance shifts across environmental gradients, spatial scales, and time [23]. Here we use “stochastic processes” to encompass drift and dispersal, while recognizing that “neutral theory” is a specific framework that assumes demographic equivalence yet still includes both components [24]. While assembly processes have been extensively examined within single habitat types, including reservoirs [13,14], rivers [25,26], wetland waters [27], and wetland soils [28,29], direct comparisons between the reservoir water and adjacent soil in coastal wetlands remain scarce. Existing comparative work has focused on riverine and estuarine systems [30,31,32], where the dispersal limitation is often stronger in soils than in aquatic habitats [30,32]. Moreover, network analysis has become a powerful tool for revealing interaction patterns among microbial taxa, offering new insights into the co-occurrence and connectivity within microbial communities [25,33]. It is now evident that the uniqueness of environmental conditions exerts a large influence on the interaction of bacterial taxa across different habitats [30,34,35].

Despite advances, several knowledge gaps remain in our understanding of abundant and rare bacterial taxa between reservoir and soil habitats. We hypothesize the following: Hypothesis 1: The habitat type (reservoir vs. soil) will significantly structure bacterial communities, with effects generally more pronounced for rare taxa given their narrower niches and higher sensitivity to environmental variation; however, rare taxa may also exhibit weaker or less stable patterns with individual environmental gradients under harsh conditions. Hypothesis 2: The balance between deterministic selection and stochastic processes differs between abundant and rare taxa in a context-dependent manner. While abundant taxa often reflect environmental selection at broad scales, they may also experience a stronger dispersal limitation, and rare taxa may be shaped by both selection and dispersal processes; thus, both groups are expected to vary along a selection–stochasticity continuum depending on the habitat characteristics and season [21,23]. Hypothesis 3: The habitat type shapes co-occurrence network properties. Relative to reservoirs, soils exhibit greater physical and chemical heterogeneity (e.g., pore size distributions, moisture and oxygen gradients, steep pH and salinity microgradients, and patchy organic substrates) and more pronounced spatial structuring. Such heterogeneity can increase local connectivity and promote modularity by concentrating edges within microhabitat-specific groups [36,37]; however, under harsh conditions, environmental filtering may reduce cross-module connectivity and thereby lower the effective network complexity despite the higher within-module cohesion.

Therefore, the objectives of this study are to (1) elucidate the differences and connectivity in the community structure (alpha and beta diversity, species turnover and exchange) of abundant and rare bacterial taxa between reservoir and soil habitats; (2) assess the assembly mechanisms governing both taxa in each habitat, explicitly considering the potential for a stronger dispersal limitation in abundant taxa and context-dependent selection in rare taxa; and (3) compare co-occurrence network structures to clarify the distinct ecological roles of abundant and rare taxa, including whether environmental filtering under harsh conditions reduces cross-module complexity while increasing modular compartmentalization. A better understanding of the assembly and interaction patterns of wetland bacterial communities is crucial for advancing the biodiversity–stability relationship and for informing conservation and management strategies for coastal wetland ecosystems.

## 2. Materials and Methods

### 2.1. Sample Collection and Physicochemical Analysis

The Beidagang Reservoir (117°11′ E–117°37′ E, 38°36′ N–38°57′ N) is located in Tianjin’s coastal Binhai New Area, adjacent to the Bohai Bay, one of the major natural wetland reserves in northern China. The reservoir covers an area of 164 km^2^, with a storage capacity of 0.50 billion m^3^ and an average water depth ranging from 2 to 3 m. The surrounding land use types primarily include wetland, forest, cropland, and industrial land [38].

**Figure 1:**
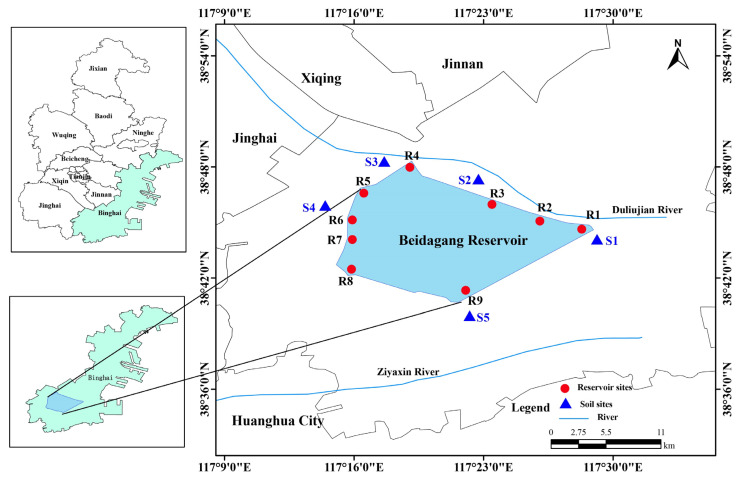
Map of sampling locations in the reservoir and adjacent soil. Red circles denote reservoir sites (R1−R9); blue triangles denote soil sites (S1−S5).

Field sampling was conducted in December 2021 in the Beidagang Reservoir and its adjacent riparian soils (Figure 1). Nine sampling sites were established at locations near the reservoir’s sluice gates. This period corresponds to the dry season, when hydrological and physicochemical conditions are relatively stable and thus comparable across sites. At each site, water samples were collected from three depths, surface (0.5 m below surface), middle (midway between surface and sediment), and bottom (0.5 m above the sediment), resulting in a total of 27 water samples. To represent the average hydro-environmental condition at each depth while ensuring sufficient biomass for downstream assays, we adopted a composite sampling strategy. For each depth, three 2 L parallel samples were collected and then combined to create a homogenized composite sample, which was transported to the laboratory without delay. Each composite water sample was subsequently divided into two portions: one for water chemistry analysis and the other for bacterial community analysis. One liter of each sample was sequentially filtered through a 200 μm mesh to remove larger particles and then through a 0.22 μm pore size membrane filter (GTTP, Millipore, Billerica, MA, USA) to capture microbial biomass. Filters were immediately stored at −80 °C until DNA extraction.

In this study, “soils” refer specifically to samples collected from the reservoir’s riparian zone, which is periodically exposed and subject to terrestrial soil development, thereby distinguishing it from submerged sediments. Five sites were selected around the reservoir for soil sampling. A total of 12 soil samples were obtained from three depth intervals: surface (0–20 cm), middle (20–40 cm), and lower (40–60 cm). However, one sample could not be obtained from the 20–40 cm layer, and two could not be obtained from the 40–60 cm layer due to field constraints. To capture small-scale spatial heterogeneity, at each site and depth, five soil cores were randomly collected and pooled at equal fresh mass to generate one composite sample for analysis. All soil samples were placed in sterile plastic bags and immediately transported to the laboratory at 4 °C. Upon arrival, a portion of each sample was stored at −80 °C for microbial community analysis, while the remainder was air-dried and pretreated for soil physicochemical measurements.

For reservoir water, environmental variables, including pH, oxidation–reduction potential (Eh), dissolved organic carbon (DOC), ammonia nitrogen (NH4^+^–N), total nitrogen (TN), total phosphorus (TP), and total dissolved solids (TDS, representing salinity), were measured following previously described protocols [4]. For soil samples, pH was measured in the soil–water slurry (1:5, soil: water, w:w) with a pH meter. Total soil salt (TSS) content was measured gravimetrically. For soil samples, pH was measured in a 1:5 (w:w) soil–water suspension using a PHS-3C pH meter (Shanghai Leici Instrument Inc., shanghai, China). Total soil salt (TSS, representing salinity) content was measured gravimetrically. Soil total organic carbon (TOC) was quantified using dichromate oxidation. Soil TN was measured by the Kjeldahl digestion method [39]. Soil TP was extracted using HF–HClO_4_ (both from Sinopharm Chemical Reagent Co., Ltd., Shanghai, China) [40] and determined by the molybdenum blue method. NH_4_^+^-N in soil was extracted with KCl solution (Sinopharm Chemical Reagent Co., Ltd., Shanghai, China) and determined by the spectrophotometric method [41]. Soil cation exchange capacity (CEC) was measured using the cobalt hexammine trichloride (Cohex) extraction–spectrophotometry method [42].

### 2.2. DNA Extraction, PCR Amplification, and Sequencing Analysis

Genomic DNA extractions from the water and soil samples were conducted using a DNeasy PowerSoil kit (Qiagen, Hilden, Germany) following the manufacturer’s instructions, respectively. To monitor potential contamination during extraction, negative controls (extraction blanks with no sample added to the kit) were processed alongside each batch of samples. Subsequently, the purity and quantity of DNA were verified by a NanoDrop 2000 spectrophotometer (Thermo Fisher Scientific, Waltham, MA, USA) and agarose gel electrophoresis, respectively. The diluted DNA was used as the template for polymerase chain reaction (PCR) amplification of bacterial 16S rRNA genes with the barcoded primers and Takara Ex Taq polymerase (Takara, Bio Inc., Osaka, Japan). PCR amplification included negative controls (no-template reactions containing all reagents except DNA) to detect potential contaminant DNA in the PCR mixture. The high-fidelity Takara Ex Taq enzyme, produced by Takara, facilitates efficient and accurate amplification. The V3-V4 hypervariable region of the bacterial 16S rRNA gene was amplified by PCR with primers 343F (5′-TACGGRAGGCAGCAG-3′) and 798R (5′-AGGGTATCTAATCCT-3′) [43,44]. PCR amplification and library preparation were carried out according to the manufacturer’s instructions and standard protocols. Purified amplicons were sequenced using the Illumina MiSeq platform at OE Biotech Co., Ltd. (Shanghai, China). Raw sequences from negative controls (extraction and PCR blanks) were processed along with samples to assess potential contamination. Sequence features detected in negative controls were flagged and subtracted from corresponding samples if present above background levels as defined in our pipeline.

Paired-end reads were then preprocessed using cutadapt (version 4.0) software to detect and cut off the adapter. After trimming, paired-end reads were filtered low-quality sequences, denoised, and merged, and chimera reads were detected and cut off using DADA2 with the default parameters of QIIME2 platform [45] to generate amplicon sequence variant (ASV) tables. Taxonomic classification of ASVs was conducted using the SILVA database (v.138) [46]. Following quality control, a total of 2,407,075 high-quality sequences from water and soil samples were retained, with individual samples ranging from 55,690 to 68,582 sequences. A total of 5175 ASVs were obtained for reservoir water, and 8934 ASVs were obtained for soil, which were used in subsequent analyses. Although sequencing depth varied among samples, rarefaction analysis demonstrated that the sequencing depth of all samples was sufficient to saturate the diversity (Figure S1). Therefore, to maximize data retention and accurately reflect microbial diversity, the original sequence counts for each sample were used for all subsequent ASV-based analyses.

To facilitate reproducible comparisons of sub-communities at the extremes of the abundance distribution, we adopt operational thresholds that are widely used in microbial ecology. Specifically, following previous studies [14,47,48], we classify ASVs within each habitat as rare when their mean relative abundance across samples is ≤0.01% and abundant when it is ≥0.1%; ASVs with intermediate values are considered moderate. These thresholds provide a consistent delineation of low- versus high-abundance assemblages while retaining ecological interpretability. To assess the impact of the arbitrary definition of abundant and rare ASVs, a multivariate cutoff-level analysis [49,50] was performed. ASVs were progressively excluded from the rarest to the most abundant, and structural similarity (Spearman correlations between Bray–Curtis distance matrices) and concordance of major ordination axes (Procrustes analysis) were quantified (Figure S2).

We also assessed the alignment of these abundance thresholds with the natural distribution of ASVs by examining abundance–frequency relationships. For each habitat, we calculated the frequency of each ASV (number of samples in which it was present) and its mean relative abundance across samples. We plotted log10(mean relative abundance) against frequency and overlaid least-squares linear fits; the 0.01% and 0.1% thresholds were indicated by horizontal dashed lines (Figure S3). We further summarized group-level characteristics, including the number and percentage of ASVs, the number and percentage of sequences, the median frequency, the interquartile range (IQR) of frequency, and the median of mean relative abundance (Table S1). This analysis confirmed that the thresholds partition ASVs into distinct regions along the abundance–frequency continuum, supporting their ecological relevance and minimizing the risk of misclassifying assembly processes.

Subsequent analysis focused primarily on abundant and rare taxa because of their distinct ecological roles and contrasting responses to environmental factors. Sequences of abundant and rare ASVs from both reservoir and soil habitats were aligned using MAFFT v7.526 [51], with an appropriate algorithm automatically selected based on dataset size and complexity. A maximum likelihood phylogeny was inferred with FastTree v2.2 under the General Time Reversible model with among-site rate heterogeneity [52]. The resulting Newick tree was midpoint-rooted and pruned to ensure a one-to-one correspondence between tip labels and the ASV table. Phylogenetic tree visualization was performed in R using the packages “ggtree” (version 3.14.0) and “ggtreeExtra” (version 1.19.990) (Figure S4).

### 2.3. Statistical Analyses

Bacterial communities were classified into four groups based on habitat (reservoir or soil) and abundance category (abundant or rare taxa). We used two complementary ordinations on Bray–Curtis dissimilarities with the “vegan” package (version 2.7.1): principal coordinates analysis (PCoA) and non-metric multidimensional scaling (NMDS). PCoA was chosen to visualize the overall habitat × taxa patterns and to report the proportion of variance explained by each axis, facilitating interpretation of the dominant gradients among the four groups. NMDS, a rank-based method, was applied to assess vertical stratification (differences across reservoir water layers and soil depths in the adjacent riparian zone) because it is robust to non-linear relationships and provides an interpretable stress value as a measure of ordination quality.

To test compositional differences, we used permutational multivariate analysis of variance (PERMANOVA) with the “adonis” function on Bray–Curtis distances to evaluate the effects of habitat, taxa abundance category, and their interaction; where relevant, pairwise contrasts were performed within habitats. Analysis of similarity (ANOSIM) was used with the “anosim” function to evaluate the significance of compositional dissimilarities among water layers and soil depths. We used permutation-based methods (PERMANOVA with restricted permutations, Mantel tests) because they are appropriate and statistically valid for unbalanced designs, and we ran a large number of permutations to ensure stable *p*-values.

Alpha diversity indices, including observed richness and Shannon–Wiener index for abundant and rare taxa, were calculated with the “vegan” package (version 2.7.1). Faith’s phylogenetic diversity (Faith’s PD) was determined using the phylogenetic tree constructed in Section 2.2, through the “pd” function in the “picante” package (version 1.8.2) [53]. Beta diversity was assessed using Bray–Curtis dissimilarity and computed via the “metaMDS” function in “vegan.” To further characterize the mechanisms underlying community differences between abundant and rare taxa in the two habitats, beta diversity values were partitioned into turnover (species replacement) and nestedness (richness difference) components, using the “beta.div.comp” function in the “adespatial” package (version 0.3.28) [54].

Reservoir samples were divided into two vertical layers: upper (surface) and deep (including both middle and bottom layers). This resulted in four groups based on habitat and taxa abundance and six groups based on three habitat strata (upper reservoir, deep reservoir, and soil) and taxa abundance. Linear Discriminant Analysis Effect Size (LEfSe) was applied to identify bacterial biomarkers across the four main groups using the “microeco” package (version 1.15.0) [4,55], with selection criteria of LDA score (log_10_) > 4.5 and *p* < 0.05. The “VennDiagram” (version 1.7.3) and “UpSetR” (version 1.4.0) packages were used to visualize shared and unique taxa among four groups and six groups, respectively.

Correlations between community structure (in both abundant and rare taxa) and environmental variables in the reservoir and adjacent soil were analyzed using Mantel tests with the “mantel_test” function in the “linkET” package (version 0.0.7.4). Canonical correspondence analysis (CCA) was conducted, as detrended correspondence analysis indicated gradients exceeding four. Permutation tests using the “envfit” function from “vegan” evaluated the statistical significance of key environmental variables shaping community composition. Furthermore, spatial autocorrelation was accounted for by integrating significant spatial eigenvectors (*p* < 0.1) derived from Moran’s eigenvector maps (MEMs) as covariates in PERMANOVA models (Bray–Curtis, 9999 permutations), allowing environmental effects to be evaluated after controlling for spatial structure (stepwise procedure in the Supplementary Materials). For families of multiple tests, *p*-values were adjusted for multiple testing using the Benjamini–Hochberg FDR method; significance annotations reflect adjusted *p*-values.

Ecological processes governing community assembly were inferred using a community-level null model framework for both abundant and rare taxa in reservoir and soil, following established protocols [22,23,56]. The β-nearest taxon index (βNTI) and Bray–Curtis-based Raup–Crick (RCbray) metrics were calculated with the “iCAMP” package (version 1.5.12) based on the phylogenetic tree. A βNTI > 2 indicates that heterogeneous selection (a deterministic process) drives community assembly, whereas a βNTI < −2 suggests dominance of homogeneous selection. Values of |βNTI| < 2 imply that stochastic processes are primarily responsible for observed phylogenetic diversity patterns. Stochasticity can be further classified with RCbray: (1) |βNTI| < 2 and RCbray < −0.95 indicate homogenizing dispersal; (2) |βNTI| < 2 and RCbray > 0.95 indicate dispersal limitation; and (3) |βNTI| < 2 and |RCbray| < 0.95 indicate “undominated” processes, such as weak selection, weak dispersal, diversification, or genetic drift [22]. Habitat niche breadths of abundant and rare taxa were estimated using the “spaa” package (version 0.2.5) [57]. Relationships between βNTI and Bray–Curtis dissimilarity in abundant and rare taxa for the reservoir and adjacent soil were assessed by Mantel tests (9999 permutations), which account for the non-independence of pairwise distances [58,59].

Co-occurrence network analysis, which included abundant, moderate, and rare taxa, was performed by calculating Spearman’s rank correlations between ASVs occurring in at least three samples within each habitat, using the “Hmisc” package (version 5.2.3). The false discovery rate (FDR) adjustment was applied to control for multiple comparisons [60]. Only robust and significant correlations (|r| > 0.8, *p* < 0.05) were retained for network construction. Network topological features, including node/edge counts, betweenness centrality, closeness centrality, eigenvector centrality, proportions of positive/negative correlations, average degree, average path length, diameter, density, clustering coefficient, degree centralization, betweenness centralization, and modularity, were calculated with the “igraph” package (version 2.1.4). Networks were visualized in Gephi (v0.10). For comparison, 1000 Erdős–Rényi random networks with matching node and edge counts were generated using “igraph” package (version 2.1.4).

Within-module connectivity (Zi) and among-module connectivity (Pi) for each node were calculated with the “microeco” package (version 1.15.0) to assign topological roles. Nodes were classified as network hubs (Zi ≥ 2.5, Pi ≥ 0.62), module hubs (Zi ≥ 2.5, Pi < 0.62), connectors (Zi < 2.5, Pi ≥ 0.62), or peripherals (Zi < 2.5, Pi < 0.62), following established criteria [61,62,63]. Keystone taxa were defined as network hubs, module hubs, and connectors [64,65]. To enable cross-habitat network comparisons, node topological properties (degree, betweenness centrality, closeness centrality, and eigenvector centrality) were normalized to percentile ranks (0 to 1) within each network. All statistical analyses were conducted in R version 4.4.2 (http://www.r-project.org, accessed on 1 December 2024).

## 3. Results

### 3.1. Community Structure and Diversity of Abundant and Rare Taxa in Reservoir and Adjacent Soil

Summary statistics for abundant, moderate, and rare taxa (ASV and sequence counts) are provided in Supplementary Table S1. Within the reservoir bacterial community, a total of 124 ASVs (2.40%) were classified as abundant taxa, accounting for 75.81% of the overall sequence abundance. In contrast, rare taxa made up the majority (4454 ASVs; 86.07% of total ASVs) but contributed only 6.81% to the total sequence abundance. In the adjacent soil, 162 ASVs (1.81%) were defined as abundant taxa, comprising 39.93% of the overall sequence abundance, while rare taxa (7313 ASVs; 86.07%) overwhelmingly dominated numerically and contributed 18.86% of the total bacterial abundance. The multivariate cutoff-level analysis showed the high structural similarity and ordination concordance between original and truncated datasets up to the substantial removal of rare (Spearman ρ > 0.99, Procrustes m > 0.99) and abundant (Spearman ρ > 0.92, Procrustes m > 0.91) ASVs (Figure S2), supporting the stability of the primary community structure across a broad range of cutoffs.

Abundance–frequency relationships further supported the ecological relevance of the adopted abundance thresholds. Both habitats showed significant positive linear relationships between frequency and abundance (reservoir: R^2^ = 0.60, *p* < 0.001; soil: R^2^ = 0.48, *p* < 0.001) (Figure S3). The 0.01% and 0.1% cutoffs aligned with natural breaks along this continuum: rare ASVs were concentrated in the low-frequency/low-abundance region, abundant ASVs were clustered at higher frequencies and abundances, and moderate ASVs formed an intermediate band. Consistently, summary statistics in Table S1 showed monotonic increases from rare to abundant taxa in both habitats for the median frequency and the median of the mean relative abundance (reservoir: 1 → 8 → 20.5; 0.0007% → 0.02% → 0.19%; soil: 1 → 3 → 7; 0.0017% → 0.02% → 0.18%). Together, these patterns indicate that the selected thresholds are data-consistent and help minimize the potential misclassification of assembly processes in the coastal wetland context.

**Figure 2:**
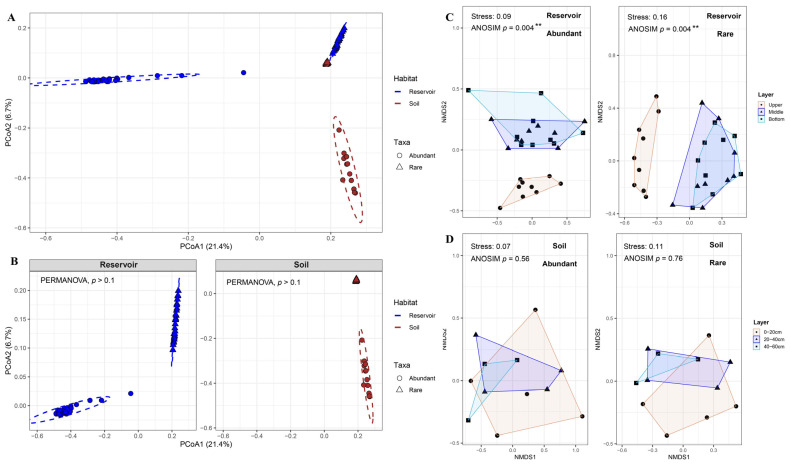
(**A**,**B**) Principal coordinates analysis (PCoA) based on Bray–Curtis dissimilarity for four groups defined by habitat (reservoir and soil) and bacterial taxa abundance (abundant and rare) (**A**) and for abundant and rare taxa within each habitat (**B**). PERMANOVA results (Adonis) indicated no significant difference. Total sample size: *n* = 78. Groups sample sizes: Reservoir Abundant (*n* = 27), Reservoir Rare (*n* = 27), Soil Abundant (*n* = 12), and Soil Rare (*n* = 12). (**C**,**D**) Non-metric multidimensional scaling (NMDS) analysis based on Bray–Curtis distances for abundant and rare bacterial taxa in the reservoir across water layers (**C**) and in the adjacent soil across different layers (**D**). Community dissimilarity among layers was assessed using analysis of similarity (ANOSIM). Reservoir layers: each layer has *n* = 9 per category (abundant, rare). Soil depths: 0−20 cm *n* = 5, 20−40 cm *n* = 4, and 40−60 cm *n* = 3 per category (abundant, rare). Significance levels: ** *p* < 0.01.

The PERMANOVA on all samples (*n* = 78) confirmed significant main effects of the habitat (reservoir vs. soil) and abundance category (abundant vs. rare), as well as a significant habitat × taxa interaction (all *p* < 0.001; Table S2). These effects were visualized using the PCoA of Bray–Curtis dissimilarities, in which samples were primarily separated by habitat with a secondary separation by the abundance category across the four groups (Figure 2A). Consistent with the PERMANOVA results, pairwise PERMANOVA comparisons within each habitat detected no significant differences between abundant and rare taxa (reservoir: *p* > 0.5; soil: *p* > 0.5; Table S3). Separate PCoA plots within the reservoir and within the soil illustrated the same pattern: within-habitat abundant vs. rare groups were only partially separated visually and did not differ significantly in statistical tests (both *p* > 0.1; Figure 2B). Together, these results indicate that the habitat is the dominant source of beta diversity, whereas within-habitat differences between abundant and rare taxa are comparatively weak.

NMDS ordinations further assessed the vertical stratification. In the reservoir, both abundant and rare communities differed among the three water layers (upper, middle, bottom; ANOSIM, *p* < 0.01), whereas no significant stratification was detected across soil depths (0–20, 20–40, 40–60 cm; ANOSIM, *p* > 0.5; Figure 2C,D). Within the reservoir, pairwise tests showed significant differences between the upper and middle layers and between the upper and bottom layers (ANOSIM and PERMANOVA, *p* < 0.05) but not between the middle and bottom layers for either abundant or rare taxa (*p* > 0.1; Table S4). Based on this pattern, we combined the middle and bottom reservoir layers as a “deep layer” for subsequent diversity analyses and compared it with the upper layer.

**Figure 3:**
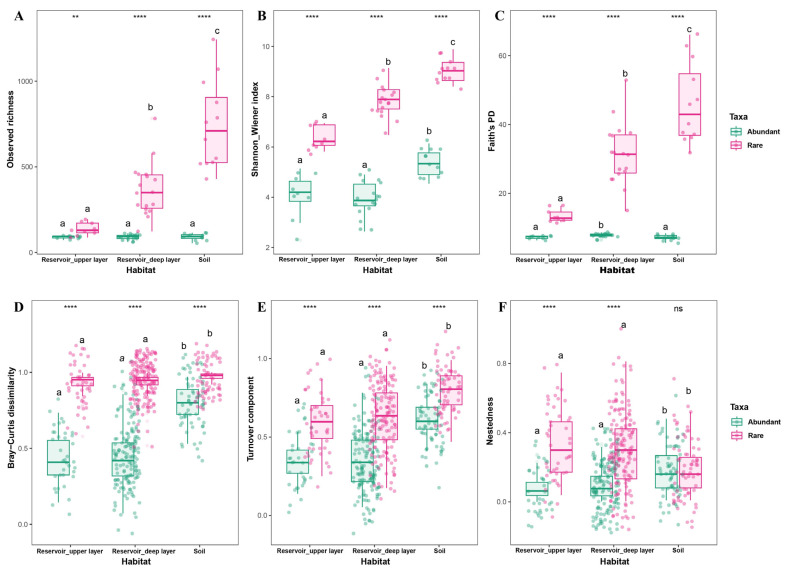
Differences in bacterial diversity of abundant and rare taxa in the upper layer and deep layer of reservoir and adjacent soils. (**A**–**C**) Alpha diversity indices: observed richness, Shannon–Wiener index, and Faith’s PD. (**D**–**F**) Beta diversity metrics: Bray–Curtis dissimilarity, turnover component, and nestedness fraction. Statistical significance was assessed using the Wilcoxon rank sum test for pairwise comparisons between abundant and rare taxa and the Kruskal–Wallis test for comparison between the upper and deep reservoir layers and soil. Significance levels between taxa are indicated as ns (not significant), ** FDR *p* < 0.01, and **** FDR *p* < 0.0001. Significance levels among habitats are indicated by letters (a, b, c), with different letters denoting significance at *p* < 0.05. Sample sizes per taxa: reservoir upper layer *n* = 9; reservoir deep layer *n* = 18; and soil *n* = 12.

Alpha diversity indices (observed richness, Shannon–Wiener index, and Faith’s PD), the beta diversity (Bray–Curtis dissimilarity), and the partitioning of beta diversity into turnover and nestedness components were used to assess bacterial diversity patterns between habitats and among abundant and rare taxa. Results indicated that rare taxa consistently exhibited significantly higher alpha diversity, beta diversity, and turnover components than abundant taxa across all habitats (Figure 3A–D). For rare taxa, soil communities displayed the highest alpha diversity values compared to the deep and upper reservoir layers. Faith’s PD of abundant taxa was comparatively similar to that of the reservoir upper layer rather than the deep layer. On average, turnover contributed the most to the beta diversity for both abundant and rare taxa (upper reservoir: 81.63% and 76.84%; deep reservoir: 65.78% and 67.52%; soil: 78.08% and 64.78%), while nestedness accounted for a smaller fraction. Furthermore, the Bray–Curtis dissimilarity, turnover, and nestedness of both abundant and rare taxa were significantly greater in the soil than in either the upper or deep reservoir layers (Figure 3D–F).

### 3.2. Community Composition of Abundant and Rare Taxa in the Reservoir and Adjacent Soil

In this study, we represented the absolute abundance of the abundant and rare bacterial groups by the total sequencing read counts (i.e., reads) obtained for each group, rather than by direct 16S rRNA gene copy numbers. Specifically, the total reads of the abundant taxa were higher in the reservoir than in the soil (upper layer: 50,025; deep layer: 45,338 vs. soil: 24,511), whereas the rare taxa in the soil had greater read counts (11,580) than those in the reservoir (upper layer: 2172; deep layer: 5237) (Figure S5). This pattern in the sequencing depth supports the trends observed in the relative abundance profiles.

**Figure 4:**
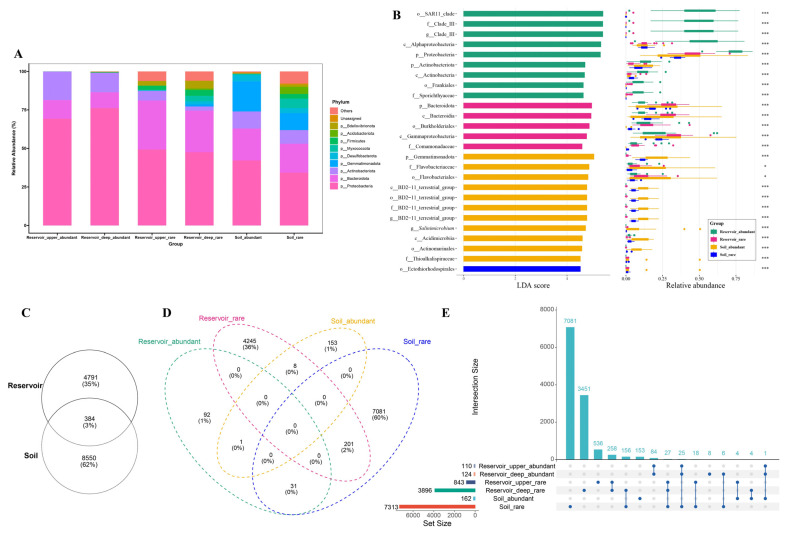
(**A**) Phylum-level composition of the top 10 abundant and rare bacterial taxa in the upper reservoir, deep reservoir, and adjacent soil. (**B**) Twenty-six biomarkers of four groups were identified with linear discriminant analysis (LDA) scores greater than 4.5, with their mean relative abundances (bars) ± standard error of the mean (error bars) (*p* < 0.05). Significance levels between taxa are indicated as * FDR *p* < 0.05 and *** FDR *p* < 0.001. (**C**–**E**) Venn and upset diagrams showing shared and unique ASVs: (**C**) between reservoir and adjacent soil; (**D**) among four groups defined by habitat (reservoir and soil) and taxa (abundant and rare); and (**E**) among six groups defined by habitat (upper reservoir, deep reservoir, and soil) and taxa.

At the phylum level (Figure 4A), Proteobacteria and Bacteroidota were predominant across all six groups. Proteobacteria were especially dominant in the abundant taxa of both reservoir layers (69.24% in the upper layer, 76.13% in the deep layer) and progressively decreased in the rare taxa from the reservoir (49.29% in the upper layer, 47.51% in the deep layer) as well as in both abundant (42.24%) and rare taxa (34.25%) from the soil. Bacteroidota showed the highest relative abundance in the rare taxa of the upper (31.82%) and deep reservoir layers (27.00%) but were less represented in the abundant reservoir taxa (12.16% in the upper, 10.37% in the deep layer). Actinobacteriota were notably more enriched in the abundant reservoir taxa (18.52% in the upper, 12.73% in the deep layer) than in the rare taxa from the reservoir, while their abundance was similar between the abundant (11.00%) and rare (8.88%) soil taxa. Gemmatimonadota were distinctive, occurring mainly in the soil’s abundant (19.55%) and rare taxa (11.02%), and were nearly absent in the reservoir (<2%). The rare taxa in soil also showed higher relative abundances of Desulfobacterota and Myxococcota compared to those in the reservoir.

Biomarker taxa distinguishing the four groups were identified by the LEfSe analysis. A taxonomic cladogram highlighted the characteristic lineages differentiating abundant and rare taxa in the reservoir and soil (Figure S6). Based on an LDA score threshold (log_10_ > 4.5, *p* < 0.05), a total of 14 biomarker taxa (9 in abundant and 5 in rare taxa) were identified for the reservoir, while 12 biomarkers were found for the soil (11 in abundant, 1 in rare taxa) (Figure 4B). In each case, the biomarker taxa in a group showed the highest relative abundance in their respective comparison.

Venn diagrams revealed that only 384 ASVs (3% of the total) were shared between the reservoir and soil habitats, underscoring a substantial divergence in the bacterial community composition. The number of shared ASVs between reservoir_abundant and soil_abundant groups (1 ASV) was much lower than that between reservoir_rare and soil_rare groups (201 ASVs). Furthermore, the abundant taxa in the reservoir shared 31 ASVs with the rare taxa of soil, while the rare taxa of the reservoir shared 8 ASVs with the abundant taxa of soil (Figure 4C), with the detailed taxonomic information of these shared ASVs listed in Table S5. Additionally, the upset diagrams revealed that the overlap of rare taxa ASVs between the deep reservoir and soil was greater than the overlap observed between the upper reservoir and soil (Figure 4D).

### 3.3. Influencing Factors and Assembly Mechanisms of Abundant and Rare Bacterial Taxa

The basic environmental characteristics of the reservoir water and adjacent soil are presented in Table S6. In the reservoir, the concentration of DOC in the middle and bottom layers was significantly higher than in the upper layer (*p* < 0.05), whereas other environmental variables exhibited no significant vertical variation (*p* > 0.05; Figure S7A). Similarly, in the adjacent soil, the pH in the 0–20 cm layer was significantly lower than in the 20–40 cm layer (*p* < 0.05), while other environmental variables did not differ significantly across soil layers (*p* > 0.05; Figure S7B). It is worth noting that salinity differed strongly between habitats. In the reservoir, the TDS of individual samples ranged from 1213 to 6080 mg/L, indicating mildly brackish conditions. In contrast, the adjacent soils were strongly saline, with the TSS in individual samples ranging from 2.02 to 31 g/kg. This pronounced water–soil salinity contrast provides a harshness gradient relevant for interpreting subsequent community–environment relationships.

**Figure 5:**
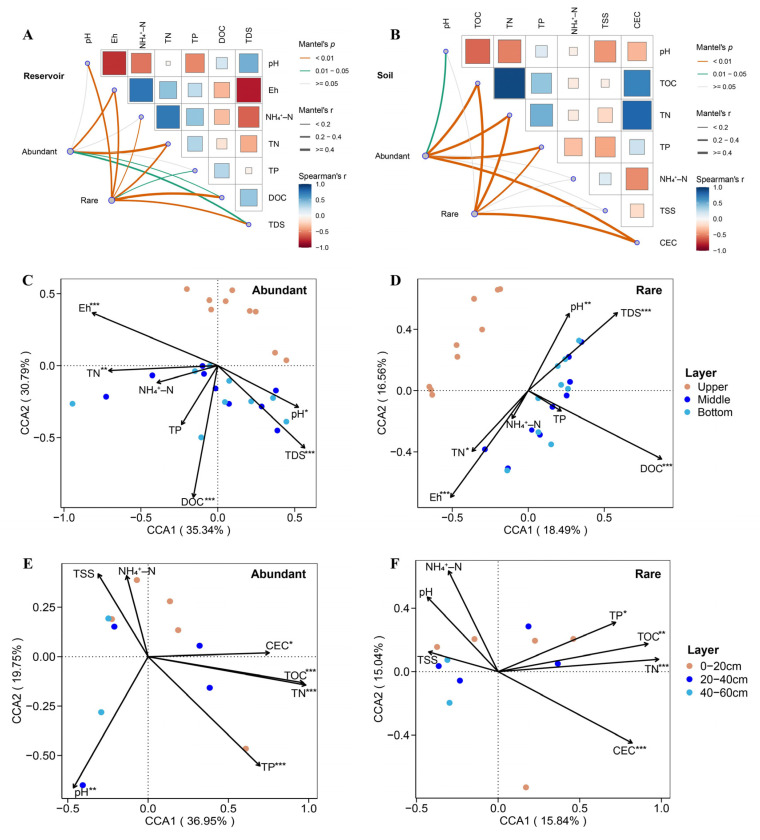
Relationships between different taxa bacterial community structures and environmental variables in the reservoir water and adjacent soil. (**A**,**B**) Mantel test examines relationships between bacterial taxa and environmental variables. Pairwise comparisons of environmental factors are shown at the top right, with color gradients representing Spearman’s correlation coefficients. Line width indicates the Mantel r statistic, and line color denotes significance based on 999 permutations. (**C**–**F**) Canonical correspondence analysis (CCA) shows the environmental variables influencing abundant and rare bacterial communities in the reservoir (**C**,**D**) and adjacent soil (**E**,**F**). Statistical significances were determined by permutation tests. Significance levels: * FDR *p* < 0.05, ** FDR *p* < 0.01, and *** FDR *p* < 0.001. Sample sizes: Reservoir water *n* = 27; adjacent soil *n* = 12 (the same samples were used for abundant and rare taxa). Arrows represent environmental variables; the arrow direction indicates the direction of the environmental gradient, and arrow length is proportional to the relative strength of that variable in explaining community composition.

To elucidate the environmental influences on bacterial communities, Mantel tests and a canonical correspondence analysis (CCA) were performed to assess the responses of abundant and rare taxa to environmental variables in both the reservoir and soil. In the reservoir, Mantel tests revealed that abundant bacterial taxa were significantly correlated with the Eh, TN, DOC, and TDS (*p* < 0.05), while rare taxa showed significant correlations with the pH, Eh, NH_4_^+^–N, TN, TP, DOC, and TDS (*p* < 0.05; Figure 5A). In the adjacent soil, abundant taxa were significantly associated with the Eh, TN, DOC, and TDS (*p* < 0.05), whereas rare taxa showed significant correlations with the pH, Eh, TOC, TN, TP, and CEC (*p* < 0.05); notably, the TOC, TN, TP, and CEC strongly influenced the structure of the rare taxa community (Figure 5B). CCA results indicated that environmental variables accounted for a greater proportion of variance in the community structure of abundant taxa compared to rare taxa, in both reservoir and soil habitats. In the reservoir, the pH, Eh, TN, DOC, and TDS were identified as the primary environmental drivers shaping both abundant and rare taxa, as supported by permutation tests (*p* < 0.05; Figure 5C,D). In the soil, the pH, TN, TP, TOC, and CEC mainly shaped the community structure of abundant taxa, while the TN, TP, TOC, and CEC were the principal drivers for rare taxa (*p* < 0.05; Figure 5E,F). In the reservoir, the abundant taxa were significantly affected by spatial processes, whereas the spatial effects on rare taxa were not significant. In the soil, no significant spatial autocorrelation was detected for the abundant community; however, in the rare taxa, the spatial terms showed marginally significant effects on the community structure, indicating that the spatial structure exerted a stronger influence on the variation in the rare community than in the abundant taxa at the study scale.

**Figure 6:**
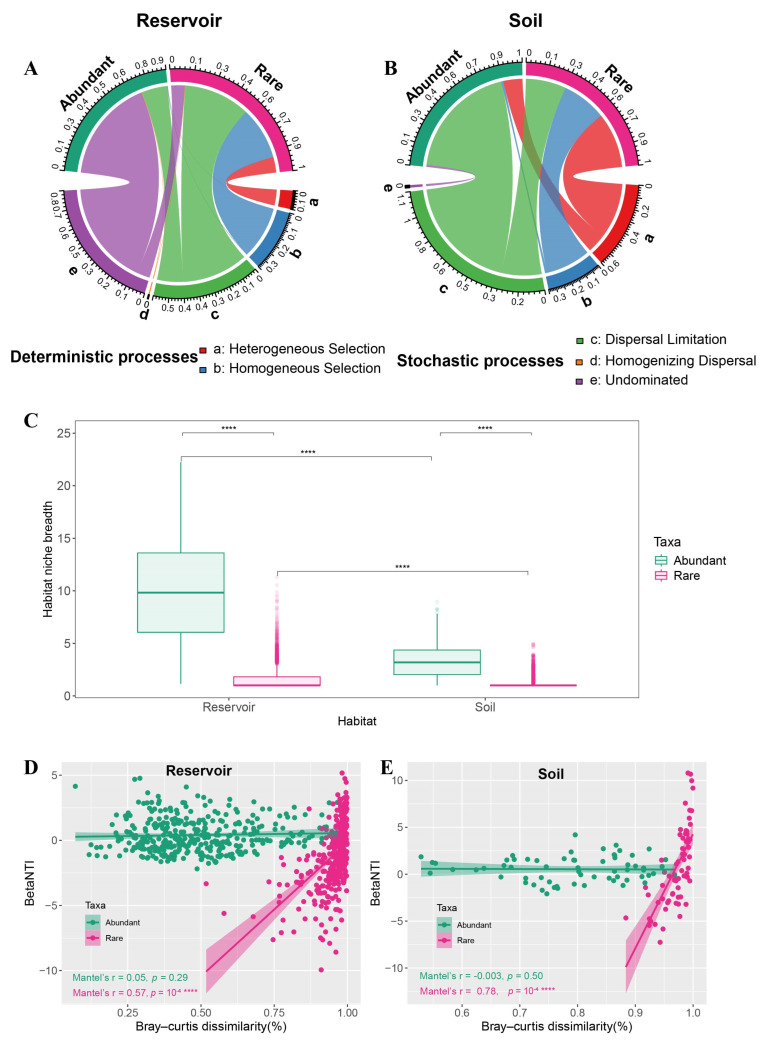
(**A**,**B**) Ecological assembly processes of abundant and rare taxa were assessed using a quantitative ecological framework based on RCBray and βNTI null models in the reservoir (**A**) and the adjacent soil (**B**). (**C**) Habitat niche breadth (Levins) of abundant and rare taxa in the reservoir and adjacent soil. Statistical comparisons between abundant and rare taxa and between reservoir and soil were conducted using the Wilcoxon test (**** FDR *p* < 0.0001). (**D**,**E**) Relationship between βNTI and Bray–Curtis dissimilarity in abundant and rare taxa for the reservoir (**D**) and adjacent soil (**E**). The associations were quantified by the Mantel tests (Mantel’s r and permutation *p*-value). Solid lines represent least-squares linear fits. Sample sizes: reservoir water *n* = 27; adjacent soil *n* = 12 (the same samples were used for abundant and rare taxa).

To further clarify the relative influence of deterministic and stochastic assembly processes, we applied a null model framework that integrates community phylogenetic relationships with abundance data. The contributions of distinct deterministic and stochastic processes for abundant and rare taxa in both the reservoir and soil were quantified using pairwise comparisons of the βNTI and RCbray (Figure S8). In the reservoir, the stochastic assembly of abundant taxa was predominately attributed to the dispersal limitation (17.38%) and undominated processes (69.80%). For rare taxa, both deterministic processes (heterogeneous selection: 10.54%, homogeneous selection: 35.33%) and stochastic processes (dispersal limitation: 44.73%, undominated: 9.12%) contributed to community assembly (Figure 6A). In the soil, the stochastic assembly of abundant taxa was mainly driven by dispersal limitation (83.33%), whereas rare taxa were shaped to a greater extent by deterministic processes (heterogeneous selection: 39.39%, homogeneous selection: 31.82%) than by dispersal limitation (28.79%; Figure 6B).

Abundant taxa showed a significantly broader niche breadth than rare taxa across both reservoir and soil habitats (Wilcoxon test, *p* < 0.0001), and the niche breadth of both groups was higher in the reservoir than in the soil (Figure 6C). Mantel tests indicated that, for rare taxa in both the reservoir and soil, the βNTI was significantly positively correlated with the Bray–Curtis dissimilarity (*p* < 0.001), whereas this relationship was not observed for abundant taxa (Figure 6D,E).

### 3.4. Co-Occurrence Networks of the Bacterial Community in the Reservoir and Soil

To elucidate interspecies interactions and the ecological roles of abundant, moderate, and rare taxa, co-occurrence networks were constructed for bacterial communities in both the reservoir and adjacent soil. The topological properties of these two networks provided insights into the complexity and structure of microbial interactions (see Table S7 for details). Compared to 1000 Erdős–Rényi random networks with the same number of nodes and edges, both empirical co-occurrence networks exhibited higher average clustering coefficients, longer average path lengths, and greater modularity. These features indicate pronounced non-random, small-world characteristics in both the reservoir and soil networks.

**Figure 7:**
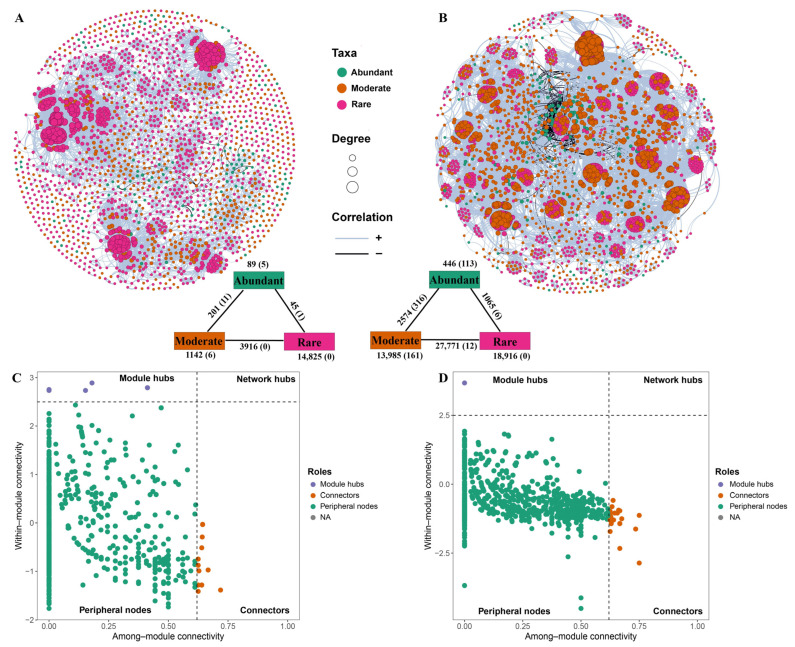
(**A**,**B**) Co-occurrence networks of bacterial communities in the reservoir (**A**) and adjacent soil (**B**), with links representing strong (Spearman’s |r| > 0.8) and significant (FDR-corrected *p*-value <0.05) correlations. The numbers outside brackets represent the numbers of positive edges, and the numbers within parentheses indicate the numbers of negative edges. (**C**,**D**) Distributions of ASVs according to within-module connectivity (Zi) and among-module connectivity (Pi) in the reservoir (**C**) and adjacent soil (**D**), illustrating the assignment of network topological roles. ASVs are classified as module hubs (Pi < 0.62 and Zi ≥ 2.5), network hubs (Pi ≥ 0.62 and Zi ≥ 2.5), connectors (Pi ≥ 0.62 and Zi < 2.5), and peripheral nodes (Pi < 0.62 and Zi < 2.5).

In the reservoir network, rare taxa comprised 66.92% of the 2143 nodes, whereas abundant taxa accounted for only 5.79% (Figure 7A). In the soil network, rare and abundant taxa represented 47.05% and 6.29% of the total 2561 nodes, respectively (Figure 7B). The soil network exhibited a substantially greater number of edges (64,757) than the reservoir network (20,218), as well as a higher average degree (50.57 vs. 18.88) and clustering coefficient (0.86 vs. 0.70). These findings indicate much tighter interspecific interactions and higher species connectivity within the soil bacterial community compared to that of the reservoir. Additionally, network modularity was greater in the soil (0.74) than in the reservoir (0.69), suggesting stronger compartmentalization in the soil bacterial community.

Potential keystone taxa were identified based on within-module connectivity (Zi) and among-module connectivity (Pi) metrics. In the reservoir network, nine module hubs and eleven connectors were recognized as keystone species (Figure 7C). Of these, three belonged to abundant taxa (ASV163, ASV187, ASV219), three belonged to moderate taxa (ASV909, ASV1729, ASV1825), and the remaining fifteen were rare taxa. These keystones spanned seven phyla and eight classes, with Proteobacteria, especially the Gammaproteobacteria and Alphaproteobacteria, being dominant. In the soil network, twenty-six keystone taxa were detected, comprising one module hub and twenty-five connectors. These included one abundant taxon (ASV436) and six rare taxa, and the remainder were classified as moderate taxa. Soil keystone taxa covered ten phyla and thirteen classes, with Proteobacteria and Bacteroidota dominating—particularly Gammaproteobacteria, Alphaproteobacteria, Bacteroidia, and Rhodothermia (Table S8).

**Figure 8:**
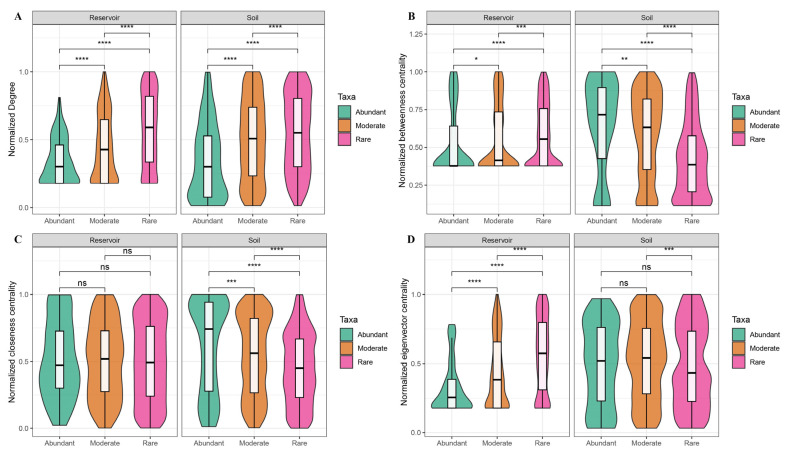
Comparison of normalized topological parameters ((**A**): degree, (**B**): betweenness centrality, (**C**): closeness centrality, and (**D**): eigenvector centrality) among abundant, moderate, and rare taxa in reservoir and soil. Topological parameters were normalized within each network to their percentile rank values. Statistical differences were assessed using the Wilcoxon rank sum test (ns: not significant, FDR *p* > 0.05, * FDR *p* < 0.05, ** FDR *p* < 0.01, *** FDR *p* < 0.001 and **** FDR *p* < 0.0001).

Node-level topological parameters for abundant, moderate, and rare taxa in both the reservoir and soil networks—including the edge/node ratio, degree, betweenness centrality, closeness centrality, eigenvector centrality, and clustering coefficient—are summarized in Table S9 and illustrated in Figure S9. Due to the overall low and dispersed values of the closeness and eigenvector centrality, all node parameters were normalized to percentile ranks within each habitat to facilitate cross-network comparisons. After normalization, rare taxa in the reservoir network exhibited a significantly higher degree, betweenness centrality, and eigenvector centrality compared to abundant taxa. In the soil network, rare taxa had higher degree values, while abundant taxa displayed higher normalized betweenness and closeness centrality than rare taxa (Figure 8).

Because the sampling occurred at a single time point, our results may be influenced by unaccounted seasonal variation or sporadic anthropogenic activities during the sampling period.

## 4. Discussion

### 4.1. The Community Structure of Abundant and Rare Taxa in the Reservoir and Adjacent Soil

In this study, reservoir water and adjacent riparian soil represent two physically connected yet ecologically distinct microhabitats within a coastal wetland ecosystem. Prior work has often compared water bodies with reservoir sediments [13], which, being permanently submerged beneath overlying water, differ fundamentally from riparian soils that undergo alternating submersion and exposure during hydrological cycles [30]. The riparian soils sampled here are periodically exposed and shaped by terrestrial soil-forming processes, making them highly sensitive to fluctuations in the water level. These dynamic conditions in riparian soils lead to frequent changes in the redox status, nutrient inputs, and energy availability, which in turn influence the structure and function of their microbial communities [29].

The comparison of the bacterial community composition revealed clear distinctions between habitats. Soil bacterial communities harbored more ASVs (8934) than reservoir water (5175), yet the overall bacterial abundance—especially that of abundant taxa—was markedly higher in reservoir water, with abundant taxa exceeding the total bacterial abundance of the soil community (Table S1). This highlights the substantial ecological and functional differentiation between reservoir water and soil. Compared with soil, reservoir water, with its greater hydrological connectivity, facilitates a broader spatial dispersal of both abundant and rare taxa, resulting in larger ecological niche breadths (Figure 6) and a greater adaptation to spatial heterogeneity. Notably, the turnover component of the community dissimilarity for both rare taxa (upper: 76.84%, deep: 67.52%) and abundant taxa (upper: 81.63%) in the reservoir exceeded that in the soil (rare: 64.78%; abundant: 78.08%). This suggests that the higher bacterial abundance and ecological turnover in the reservoir lead to greater spatial heterogeneity in the community composition.

The PCoA with PERMANOVA further confirmed that the habitat was a key determinant of the bacterial community structure, with a significant interaction between the habitat and taxon abundance (Table S2). To provide an independent validation beyond ordination, we examined how environmental variables structured communities within each habitat using Mantel tests and the CCA. Distinct drivers emerged between habitats: in the reservoir, both abundant and rare taxa were structured chiefly by salinity (TDS), nutrients, and physicochemical conditions (e.g., Eh, pH), whereas in the soil, the nutrients and CEC dominated (Figure 5). Together, these results provide independent, mechanistic support for the observed habitat effect. This convergence across ordination- and constraint-based analyses is consistent with Hypothesis 1 regarding strong habitat structuring.

In parallel, diversity patterns further underscore habitat distinctiveness. The alpha diversity (Shannon–Wiener index, Faith’s PD) and beta diversity (Bray–Curtis dissimilarity) for both abundant and rare taxa were consistently higher in soil compared to reservoir water. This elevated diversity in soil microbial communities is likely due not only to the presence of resident microbes but also to the continual exchange of microbes between soil microhabitats and their surrounding environments [32,66]. These findings are consistent with previous studies reporting that soil harbors greater microbial diversity than aquatic environments [30,31,32]. Nevertheless, such comparisons should consider system-specific attributes (e.g., salinity gradients, hydrologic connectivity, and tidal-induced wetting–drying in our coastal setting), thereby supporting Hypothesis 1, which states that soil and aquatic habitats maintain distinct and highly diverse bacterial assemblages.

The spatial stratification within the reservoir revealed clear depth-related differences in both abundant and rare communities (ANOSIM/PERMANOVA; Figure 2C; Table S4). For abundant taxa, Faith’s PD was significantly higher in the deep layer than in the surface layer, and the deep water diversity more closely resembled that of the soils than of surface waters. For rare taxa, alpha diversity indices were also significantly higher at depth, driven primarily by the far greater number of rare ASVs in the deep layer compared with the surface (3896 vs. 843; Figure 4D). Consistently, the relative abundances of Proteobacteria and Actinobacteriota decreased with depth, whereas Bacteroidota increased, across both abundant and rare communities (Figure 4A).

We interpret these depth-related patterns as the product of a composite gradient rather than spatial separation alone. Depth typically integrates co-varying environmental changes, including temperature, light penetration, dissolved oxygen, and hydrostatic pressure. Because high-resolution profiles of these variables were not available in this study, we refrain from attributing the observed patterns primarily to spatial heterogeneity. The observed resemblance between deep water and soil diversity may reflect multiple processes, potentially including hydrological or biogeochemical linkages (e.g., episodic runoff inputs or root-mediated exchange), alongside other depth-related environmental filters.

Given the unique characteristics of soil as a microhabitat, its bacterial community is frequently shaped by the rhizosphere effects [30]. In the present study, the relative abundances of Gemmatimonadota among abundant taxa and Desulfobacterota and Myxococcota among rare taxa was significantly higher in the soil than in the reservoir. Both Gemmatimonadota and Myxococcota have been reported as prominent members in coastal freshwater Phragmites wetlands [67]. Gemmatimonadota is also recognized as a dominant component of soil bacterial communities, with a higher abundance in soil than in freshwater environments, and is often associated with plants and the rhizosphere [68,69]. Consistent with these findings, Gemmatimonadota emerged as the top biomarker for abundant taxa in our study. Desulfobacterota, by contrast, are primarily associated with anaerobic sulfate reduction in soil and play crucial roles in the sulfur cycle of lagoon and estuary wetlands [67,70]. In the specific context of our saline, tidally influenced system, we infer that the rhizosphere carbon supply coupled with alternating anaerobic/microoxic conditions jointly structures the ecological niches of these phyla. This mechanism is system-specific relative to non-saline inland soils [71] or other coastal wetlands [68,69], where salinity-driven constraints and tidal wet–dry cycling are absent or attenuated.

Our findings also highlight cross-habitat microbial connectivity and the habitat dependence of rarity and abundance states. Specifically, 201 rare ASVs were shared between the reservoir and soil, 31 ASVs were abundant in the reservoir but rare in the soil, and 8 ASVs were rare in the reservoir but abundant in the soil (Table S5). These results indicate dynamic ecological connectivity between the two habitat systems and show habitat-dependent status shifts (rare ↔ abundant) within the same lineages. These observed habitat-dependent exchanges between abundant and rare taxa carry several ecological implications: (1) different bacterial taxa display habitat-specific responses to environmental heterogeneity, thereby enhancing functional diversity and ecological resilience; (2) rare taxa present in the reservoir can become abundant in the soil, supporting the concept of the rare biosphere acting as a microbial seed bank that enables rare lineages to become dominant under favorable conditions [16]; (3) both deterministic processes (e.g., habitat-specific environmental selection) and stochastic processes (e.g., cross-habitat dispersal) jointly maintain and reshuffle species across habitats; and (4) such microbial exchanges facilitate functional redundancy and resilience within communities, thereby strengthening the coupling of biogeochemical processes between wetland soils and aquatic ecosystems. Hydrological connectivity can mitigate the dispersal limitation, especially for abundant taxa (supporting Hypothesis 2), whereas the observed rare-to-abundant shifts illustrate the seed bank function of the rare biosphere under habitat-specific selection.

### 4.2. Influencing Environmental Factors and Assembly Mechanisms of Abundant and Rare Taxa in Reservoir Water and Adjacent Soil

In this study area, the strong hydrological connectivity between the reservoir water and groundwater, combined with the common meteorological recharge, has led to seawater intrusion into the reservoir, resulting in the marked salinization of the reservoir water [4,72]. The adjacent wetland soils display severe salinization [73], consistent with our measurements showing strongly saline conditions (TSS in individual samples: 2.02–31 g/kg), in contrast to the mildly brackish reservoir (TDS: 1213–6080 mg/L). Our analyses revealed that, in the reservoir, both abundant and rare taxa were significantly influenced by the nutrient variables (DOC, TN), TDS, Eh, and pH. In contrast, in the soil only nutrient variables (TOC, TN, TP) and the CEC significantly affected abundant and rare taxa. The previous research on reservoirs and lakes has demonstrated that salinity is a key factor shaping the microbial community composition [56,74,75], which supports our findings. Similarly, many studies have shown that salinity acts as an environmental filter for bacterial communities in coastal wetlands and saline gradient soils [76,77,78,79]. However, in our study, neither abundant nor rare taxa in soil were significantly correlated with the TSS, likely because the soil salinity was relatively homogeneous across samples at the sampled scale (Figure S3) despite being strongly saline overall and thus could not explain the within-habitat variation in the soil bacterial community.

With regard to community assembly mechanisms, our results showed that stochastic processes primarily governed the assembly of abundant taxa in both the reservoir and soil. In the reservoir, rare taxa were shaped by a combination of deterministic and stochastic processes, whereas in the soil, deterministic selection (71.21%) overwhelmingly dominated the assembly of rare taxa, compared to undominated stochastic processes (28.79%) (Figure 6A,B). This pattern is consistent with the strong saline soil conditions acting as a potent environmental filter that narrows the viable niche space for rare, specialist taxa. The dominance of stochastic processes in shaping abundant taxa is consistent across multiple systems, e.g., source water reservoirs [13], the wetland system [27], estuary areas of the lake [26], plain river networks [25], and coastal wetland soils [28], despite differences in the hydrodynamics, salinity regimes, and disturbance frequency among them. In contrast, rare taxa tend to be more system-dependent in aquatic environments, whereas in soils, environmental selection is typically stronger [28,31,71]—a pattern that may be further reinforced in our coastal wetland by the salinity pulses, high connectivity, and tidal wetting–drying.

Examining the association between the βNTI and Bray–Curtis dissimilarity offers additional perspective on the ecological processes structuring microbial communities. A strong positive correlation indicates that environmental filtering concurrently structures phylogenetic and taxonomic compositions, implying stronger deterministic control, whereas a weak or non-significant correlation is more consistent with stochastic dynamics [21,23]. In our study, rare taxa in both the reservoir and soil exhibited significant positive correlations between the βNTI and Bray–Curtis dissimilarity (Figure 6D,E). The Mantel correlation coefficient (r) was higher in the soil than in the reservoir (0.78 vs. 0.57; both *p* < 0.001). To communicate the effect size magnitude, we adopted commonly used thresholds for correlation coefficients [80], treating ∣r∣ ≥ 0.7 as strong and 0.4 ≤ ∣r∣ < 0.7 as moderate, adapted here for the matrix-level Mantel‘s r. Thus, for the soil the correlation was strong, and for the reservoir it was moderate. Consistently, the proportion of deterministic processes quantified by the null model for rare taxa was higher in the soil (74.07%) than in the reservoir (45.87%). Together with the harsh salinity in soils, these results indicate tighter coupling between the phylogenetic turnover and taxonomic dissimilarity under stronger environmental filtering.

Consistent with the context-dependent expectations of Hypothesis 2, our winter sampling revealed stochastic processes dominating the abundant taxa and deterministic selection and more strongly structuring rare taxa, particularly in soils. This pattern is interpreted as a consequence of the harsh, strongly saline soil conditions that generate greater microhabitat heterogeneity and stronger redox and salinity microgradients relative to the reservoir water column during winter. Such heterogeneity can impose narrow niche constraints on rare taxa, thereby enhancing the deterministic selection, while abundant, generalist taxa may be more influenced by the dispersal limitation and undominated processes. These findings are consistent with reports that the balance between selection and stochasticity can be inverted across habitats and taxa depending on the environmental context and niche breadth [27,28]. Furthermore, these findings underscore the importance of habitat-specific and seasonal considerations when evaluating assembly mechanisms.

The CCA revealed that environmental variables explained a greater proportion of the compositional variation in abundant taxa than in rare taxa across both habitats. This does not contradict the expectation that rare taxa may show weaker and less stable patterns with single gradients, particularly under harsh conditions, even when the selection is strong at the community level. At first glance, this seems to conflict with the null model inference about assembly mechanisms. However, it is important to note that the CCA quantifies the fraction of the total community variance attributed to environmental factors. Because abundant taxa are dominant and contribute the most to the total community variation, the explanatory power of environmental variables is proportionately higher. In contrast, rare taxa contribute little to the total variance and, despite their higher sensitivity to environmental change, their variation is less well captured by the CCA. From the perspective of assembly mechanisms, abundant taxa, with their broad distribution and wide ecological niches, are less affected by environmental filtering and are more likely to be governed by stochastic processes. Rare taxa, often being “environmental specialists”, are highly sensitive to environmental gradients and are more strongly regulated by deterministic processes such as environmental selection. Thus, the two analyses are not contradictory; they emphasize different ecological perspectives: one on the composition of variance and the other on the underlying drivers. This ecological disparity reflects a strategic divergence in the environmental response between abundant and rare bacterial lineages.

We recognize that assembly mechanisms can vary with the spatial scale (local vs. regional processes) and season and that evolutionary relationships shape these inferences. Our sampling captures a single spatial scale and time window, which limits direct tests of regional processes and seasonality. Accordingly, inferences about regional dispersal and seasonal shifts are interpreted with caution. Future work will extend this design to multiple spatial grains and seasons to directly test scale- and season-dependent changes in the process balance and to validate the robustness of the patterns reported here.

### 4.3. The Ecological Role of Abundant and Rare Taxa in the Network of the Reservoir and Adjacent Soil

Despite employing identical network construction methods, the co-occurrence network structures of bacterial communities differed markedly between the reservoir and soil, highlighting that environmental heterogeneity is a principal driver of these differences. Similar findings have been reported in other habitat comparisons, where the unique environmental features of each system strongly shape microbial interactions [30,34,35]. Such inter-taxa interactions substantially contribute to community resilience by buffering environmental disturbances [81], although this linkage is inferential in our context. Given our coastal setting (salinity pulses, hydrologic connectivity, and tidal wetting–drying), interpretations of the network topology should be treated as system-dependent. These habitat-driven structural differences directly support Hypothesis 3, which anticipates the network divergence between the reservoir and soil under contrasting environmental heterogeneity and harsh conditions.

Notably, the soil bacterial network was characterized by a higher edge density, average degree, clustering coefficient, and shorter average path length compared to the reservoir, indicating a more densely connected and interactive community. This denser networking, by promoting the active decomposition of soil organic carbon, may enhance the carbon utilization efficiency [33]. Moreover, the higher modularity observed in the soil network signifies greater ecological compartmentalization and the formation of discrete functional modules, which are commonly linked to environmental filtering and resource partitioning [36,37]. These highly structured modules further corroborate our findings that rare taxa in soil are subject to pronounced environmental selection (Figure 6A,B). Higher modularity is often associated with greater resilience in network theory and empirical studies and thus may indicate increased resilience; however, this inference is indirect and may vary with system-specific drivers (e.g., salinity pulses and hydrologic connectivity in coastal wetlands), as framed in Hypothesis 3.

Within ecological networks, keystone species maintain the overall structure and function of microbial communities through their extensive connections with other taxa [82,83]. In the present study, nineteen keystone species were identified in the reservoir network, functioning mainly as module hubs and connectors, whereas a larger number (26) were found in the soil, predominantly serving as connectors linking different modules [84]. The predominance of connectors as keystone taxa in soil, reminiscent of patterns found in rhizosphere environments [85], supports Hypothesis 3, which states that the habitat type shapes co-occurrence network properties, with soils exhibiting greater connectivity within modules and higher modularity overall.

In the reservoir, rare taxa contributed the majority of keystone species (15 out of 19) and exhibited markedly higher values for all major network topological parameters compared to abundant taxa (Table S9). These results underscore the central role of rare taxa in stabilizing bacterial communities within aquatic environments, corroborating previous studies in reservoir systems [13,14]. In contrast, within the soil network, abundant taxa exhibited the highest betweenness and closeness centrality (Figure 8), indicating a primary role in intermodular connectivity and core network influences. These contrasts further underscore the context dependence in ecological roles across habitats (as outlined in Hypothesis 2), wherein rare taxa stabilize aquatic networks while abundant taxa concentrate the intermodular influence in soils.

Notably, although moderate taxa comprised only 16.33% of ASVs in the soil, their total abundance (41.21%) surpassed that of the abundant taxa (39.93%). In the soil network, moderate taxa accounted for 46.66% of all nodes—second only to rare taxa at 47.05%—and contributed approximately 73% of the keystone species. While the edge/node ratio, degree, and clustering coefficient of moderate taxa were slightly less than those of rare taxa, their normalized betweenness centrality, closeness centrality, and eigenvector centrality were notably higher, highlighting their critical role in network cohesion. Furthermore, moderate taxa exhibited a pivotal bridging function, forming frequent links between abundant and rare taxa within the soil microbial network (Figure 7B). Collectively, these findings indicate an important—previously underappreciated—role of moderate taxa in maintaining a high connectivity and stability potential in soil networks. This pattern is further reflected by the more intricate associations and ecological roles of the three abundance groups in soil compared with the reservoir. It is worth noting that previous studies have classified soil bacterial communities directly into “core” and “non-core” taxa to analyze their functional roles [9]. Exploring more refined and ecologically meaningful approaches for defining taxon abundance groups remains an important direction for future research, especially when comparing patterns across diverse habitats.

### 4.4. Limitations of This Study

We used pooled samples for water and riparian soil to secure biomass and represent site–depth means. Pooling can bias results by (i) averaging parallel grabs/cores, which dilutes variability, attenuates extremes, and reduces the detection of rare or patchy taxa, and (ii) smoothing micro-scale heterogeneity, potentially shifting the community structure. To limit these effects, we applied equal-volume/mass pooling, thorough homogenization, randomized combining, same-day processing, strict cold chain handling, and field/filtration blanks. These steps improve reproducibility and approximate site-level means, but some bias likely remains, particularly for low-abundance or localized features. Future work should analyze some non-pooled replicates, add replications, or use statistical deconvolution.

In addition to pooling-related constraints, the unequal sample sizes between water and soil habitats may reduce statistical power for the less-sampled habitat, inflate uncertainty in diversity and network metrics, and bias cross-habitat comparisons. Deeper soil layers (20–40 cm, 40–60 cm) could not be collected at some sites due to a high water table and compaction. Thus, our soil inferences primarily pertain to the surface horizon. To mitigate potential biases from unequal group sizes, we used permutation-based frameworks that are valid under unbalanced designs (see Section 2). Nonetheless, the power remains lower for the smaller group. Although consistent pipelines and normalization were applied, unbalanced designs increase the risk of type II errors and can affect estimates of the co-occurrence centrality and modularity. Future studies should adopt balanced sampling schemes and, where imbalance is unavoidable, use modeling frameworks robust to an unequal n (e.g., hierarchical or mixed-effects models or permutation tests).

The sampling was conducted at a single time point, which precludes the temporal replication and limits inferences about seasonal variability. Consequently, potential seasonal dynamics and transient anthropogenic influences during the sampling window (e.g., short-term discharges or reservoir operations) cannot be ruled out. To capture temporal variability and distinguish persistent habitat signals from episodic disturbances, future work should implement multi-season or multi-year sampling—ideally comprising fixed-interval monitoring and event-triggered campaigns.

Methodologically, our reliance on 16S rRNA gene amplicon sequencing provides a robust taxonomic resolution but constrains direct functional inferences. This approach limits our ability to resolve metabolic capabilities and gene expression and therefore to link compositional patterns to trait- or pathway-level processes across abundance categories. Future investigations should integrate multi-omics approaches together with multi-season sampling to connect the community composition with the gene content and expression and to elucidate how abundant and rare taxa collectively contribute to ecosystem functions in both reservoir and soil communities.

Despite these limitations, the present study provides a coherent baseline for future, multi-season, and functional investigations of abundant and rare bacterial taxa across coupled aquatic–terrestrial habitats.

## 5. Conclusions

This case study, based on a single reservoir–riparian system sampled during winter, revealed clear differences in bacterial community structures between reservoir water and adjacent wetland soil. The reservoir water showed a higher total bacterial abundance but lower taxonomic richness and diversity relative to soils. Environmental correlates of the community composition appeared to be habitat-specific: salinity (i.e., saline conditions) and nutrient availability were most influential in the reservoir, whereas nutrient variables and the cation exchange capacity were more strongly associated with soil communities. Signals of community assembly also diverged in a context-dependent manner: stochastic processes predominated for abundant taxa in both habitats, whereas deterministic environmental selection appeared stronger for rare taxa, particularly in soils. Network analyses further indicated that, in this wintertime saline–coastal context, soil bacterial communities had greater connectivity and modularity than reservoir communities, suggesting more complex association patterns and ecological compartmentalization in soils. However, under strongly saline conditions, such complexity can be reshaped by reducing cross-module connectivity via intensified environmental filtering. Together, these findings suggest that, in this system and season, abundant and rare taxa and habitat types (water vs. soil) are associated with contrasting environmental drivers, assembly tendencies, and network properties. While these results provide system-specific insights into microbial community organization within this reservoir–riparian interface, broader generalizations should be made cautiously and await multi-season and multi-site investigations, ideally integrating higher-resolution functional approaches (e.g., shotgun metagenomics and metatranscriptomics).

## Data Availability

The obtained sequences were deposited in the National Center for Biotechnology Information (NCBI) under the Bio Projects ID: PRJNA1168442 (available at https://www.ncbi.nlm.nih.gov/bioproject/PRJNA1168442) and PRJNA1302095 (available at https://www.ncbi.nlm.nih.gov/bioproject/PRJNA1302095).

## Supplementary Materials

The following supporting information can be downloaded at https://www.mdpi.com/article/10.3390/biology14091291/s1, Method: Spatial autocorrelation analysis and spatial variable processing. Figure S1: Rarefaction curve of bacterial community Shannon index reaches saturation stage with increasing sequencing depth. Figure S2: Multivariate cutoff level analysis for the reservoir (A–C) and soil (D–F) communities based on the sequential removal of ASVs from rare to abundant. Panels: (A,D) Number of Remaining ASVs; (B,E) Structural similarity, measured as non-parametric Spearman rank correlations between Bray–Curtis distance matrices of the original and truncated communities; (C,F) Ordination concordance, quantified as Procrustes similarity of principal coordinates. The blue dashed lines indicate the proportion of ASVs classified as rare at a 0.01% mean relative abundance threshold; the blue dashed lines indicate the proportion of ASVs classified as abundant at a 0.1% mean relative abundance threshold in each habitat. Figure S3: Abundance-Frequency relationships of abundant, moderate, and rare ASVs in reservoir and soil habitats. Each point represents an ASV. The solid black lines show least-squares linear fits on the log10 scale; shaded areas indicate 95% confidence intervals. Horizontal dashed lines mark the abundance thresholds at 0.01% and 0.1%. R^2^ is the coefficient of determination for the linear regression, and *p* denotes the significant level. Figure S4: Phylogenetic tree of abundant and rare ASVs in reservoir and soil habitats. Figure S5: Phylum-level composition of the top 10 abundant and rare bacterial taxa based on sequence counts (reads) across the upper and deep reservoir layers and adjacent soils. Figure S6: Taxonomic cladogram from Linear discriminant analysis Effect Size (LEfSe) analysis identifying significant biomarker taxa in bacterial communities across four groups: reservoir-abundant, reservoir-rare, soil-abundant, and soil-rare. Figure S7: The vertical variation in environmental factors in the reservoir (A) and soil (B). The statistical differences were evaluated among layers utilizing the Kruskal–Wallis test and between layers, utilizing the Wilcoxon rank sum test (ns, not significant, * FDR *p* < 0.05, **** FDR *p* < 0.0001). Eh: oxidation-reduction potential TDS: total dissolved solids; TN: total nitrogen; TP: total phosphorus; DOC: dissolved organic carbon; Eh, oxidation-reduction potential, TSS: total soluble salts, TOC: total organic carbon, CEC: cation exchange capacity. Figure S8: β-nearest taxon index (βNTI) and Bray–Curtis-based Raup–Crick (RCbray) analyses of abundant and rare taxa between reservoir water and soil. Comparisons between abundant and rare taxa, between reservoir and soil with Wilcoxon test (ns: not significant, *** FDR *p* < 0.001, **** FDR *p* < 0.0001). Figure S9: Comparison of non-normalized topological parameters (degree, betweenness centrality, closeness centrality, and eigenvector centrality) among abundant, moderate, and rare taxa in reservoir (A) and soil (B). Statistical differences were assessed using the Kruskal–Wallis’s test and Wilcoxon rank sum test (ns: not significant, * FDR *p* < 0.05, ** FDR *p* < 0.01, *** FDR *p* < 0.001, **** FDR *p* < 0.0001). Table S1: Identification and information of abundant, moderate, and rare ASVs in reservoir and soil habitats. Description of bacterial ASV datasets based on exact sequence variants and frequency of each ASV. Table S2: Results of PERMANOVA test for effects of habitat (reservoir vs. soil) and taxa abundance types (abundant vs. rare) on bacterial community structure based on Bray–Curtis dissimilarity. Statistical significance: *** *p* < 0.001. Total sample size: 78. Sample sizes: Reservoir Abundant (*n* = 27), Reservoir Rare (*n* = 27), Soil Abundant (*n* = 12), Soil Rare (*n* = 12). Table S3: Pairwise PERMANOVA comparisons of bacterial community structure among groups defined by habitat (reservoir, soil) and taxa abundance type (abundant, rare), based on the Bray–Curtis dissimilarity. The p-values were adjusted for multiple comparisons using the Benjamini–Hochberg method. Statistical significance levels: ns, not significant; * *p* < 0.05. Table S4: Dissimilarity test of abundant and rare taxa in the reservoir water by ANOISM (analysis of similarity), and PERMANOVA (permutational multivariate analysis of variance) based on Bray–Curtis dissimilarity matrix between different water layers. Each water layer includes 9 samples for abundant taxa and 9 samples for rare taxa. Significance codes reflect adjusted p-values: * FDR *p* < 0.05; ** FDR *p* < 0.01. Table S5: Taxonomic information of shared ASVs between reservoir_abundant and soil_abundant, reservoir_abundant and soil_rare, reservoir_rare and soil_abundant. Table S6: Environmental factors of three water layers (upper, middle, and bottom) in 27 samples, of three layers (0–20 cm, 20–40 cm, and 40–60 cm) of adjacent soil in 11samples, presented as mean ± standard deviation. Table S7: Comparison of topological properties for the empirical co-occurrence networks of bacterioplankton communities in the reservoir water and soil and their associated random networks. Table S8: Taxonomic information of keystone taxa from the networks in the reservoir water and soil. Table S9: Node level topology characteristics of abundant, moderate, and rare taxa in the bacterial networks of reservoir and soil.

## Abbreviations

The following abbreviations are used in this manuscript:

Eh	Oxidation-reduction potential
TDS	Total dissolved solids
DOC	Dissolved organic carbon
TN	Total nitrogen
TP	Total phosphorus
NH_4_^+^–N	Ammonia nitrogen
TSS	Total soil salt
TOC	Total organic carbon
CEC	Cation exchange capacity
PCR	Polymerase chain reaction
ASV	Amplicon sequence variant
PCoA	Principal coordinates analysis
PERMANOVA	Permutational multivariate analysis of variance
NMDS	Non-metric multidimensional scaling
ANOSIM	Analysis of similarity
Faith’s PD	Faith’s phylogenetic diversity
LEfSe	Linear discriminant analysis effect size
CCA	Canonical correspondence analysis
βNTI	β-nearest taxon index
RCbray	Bray–Curtis-based Raup–Crick
MEMs	Moran’s eigenvector maps
