# Supplementary material for: Contrasting Assembly and Network Roles of Abundant and Rare Bacteria in Reservoir and Soil Habitats
Source: Biology (Basel). 2025 Sep 18;14(9):1291. doi: 10.3390/biology14091291 (PMC12467908; doi:10.3390/biology14091291)
Supplement: Supplementary file 1 [file biology-14-01291-s001.zip › biology-3834100-supplementary.pdf]

Journal: Biology

Appendix A Supplementary data

Contrasting Assembly and Network Roles of Abundant and Rare Bacteria in Reservoir and Soil Habitats

Cuixia Zhang [1,2,3,4], Haiming Li [1,2,3,4]*, Mengdi Li [1,2,4], Sihui Su [1,3,4], Han Xiao [5], Xiaodong Zhang [1,4] and Qian Zhang [4]

1 College of Marine and Environmental Sciences, Tianjin University of Science and Technology, Tianjin 300457, China; zhangcuixia@tust.edu.cn (C.Z.); limengdi@tust.edu.cn (M.L.); susihui@tust.edu.cn (S.S.); xiaodongzhang521@tust.edu.cn (X.Z.)

2 Key Laboratory of Marine Resource Chemistry and Food Technology, Ministry of Education, Tianjin 300457, China

3 Tianjin Key Laboratory of Marine Resources and Chemistry, Tianjin 300457, China

4 Binhai Laboratory of Groundwater utilization and Protection, Tianjin University of Science and Technology, Tianjin 300457, China; zq15536363317@163.com

5 Chinese Research Academy of Environmental Sciences, Beijing 100012, China; hxiao0716@163.com

* Correspondence: lhm@tust.edu.cn

----

* Corresponding author: Haiming Li (lhm@tust.edu.cn);

Tel.: +86 022 60600359; Fax. +86 022 60600359

First author: Cuixia Zhang (zhangcuixia@tust.edu.cn)

Address: Tianjin University of Science & Technology, No 9,13th Avenue, TEDA, Tianjin, P.R. China, 300457

This document includes:

Supplementary Methods; Figure S1 to S9; Table S1 to S9

Supplementary Methods

Spatial autocorrelation analysis and spatial variable processing

To assess the influence of spatial structure on community variation and control for spatial autocorrelation, we extracted spatial variables using Moran's eigenvector maps (MEM). We controlled spatial autocorrelation by incorporating spatial covariates (depth factor and MEM vectors derived from projected coordinates) into PERMANOVA models and quantified pure environmental effects using marginal-effects tests (by = "margin"). The stepwise PERMANOVA procedure was as follows:

(1) Based on the geographic coordinates (x, y) of sampling points, we constructed a spatial weighting matrix (inverse-distance or inverse-distance-squared weighted), performed eigen decomposition, and retained MEM variables associated with positive eigenvalues (MEM1, MEM2, …).

(2) We first ran a "space-only" model (comm ~ depth_factor + full MEM set, Bray–Curtis distance, 9,999 permutations, adonis2, by = "margin") to screen marginally significant MEM terms (threshold p<0.1), thereby avoiding model oversaturation due to small sample size.

(3) We then incorporated the screened MEM together with environmental variables (e.g., TDS, TN, TOC, CEC, TP) into a mixed model (comm ~ depth_factor + environment + screened MEM) to compare the independent effects of environmental variables after controlling for spatial structure.

(4) For robustness, we further evaluated the "pure environmental effect" within a conditioning framework (comm ~ environment + Condition (depth_factor + screened MEM)) to verify the significance of key environmental factors after spatial control.

The number of permutations was set uniformly to 9,999. When sample size was limited, we checked residual degrees of freedom to avoid model oversaturation. Prior to modeling, we verified rank/residual degrees of freedom to ensure n−rank>0 and prioritized retaining a limited number of environmentally meaningful variables to improve statistical power. All analyses were conducted in R (vegan package).

Supplementary Figures

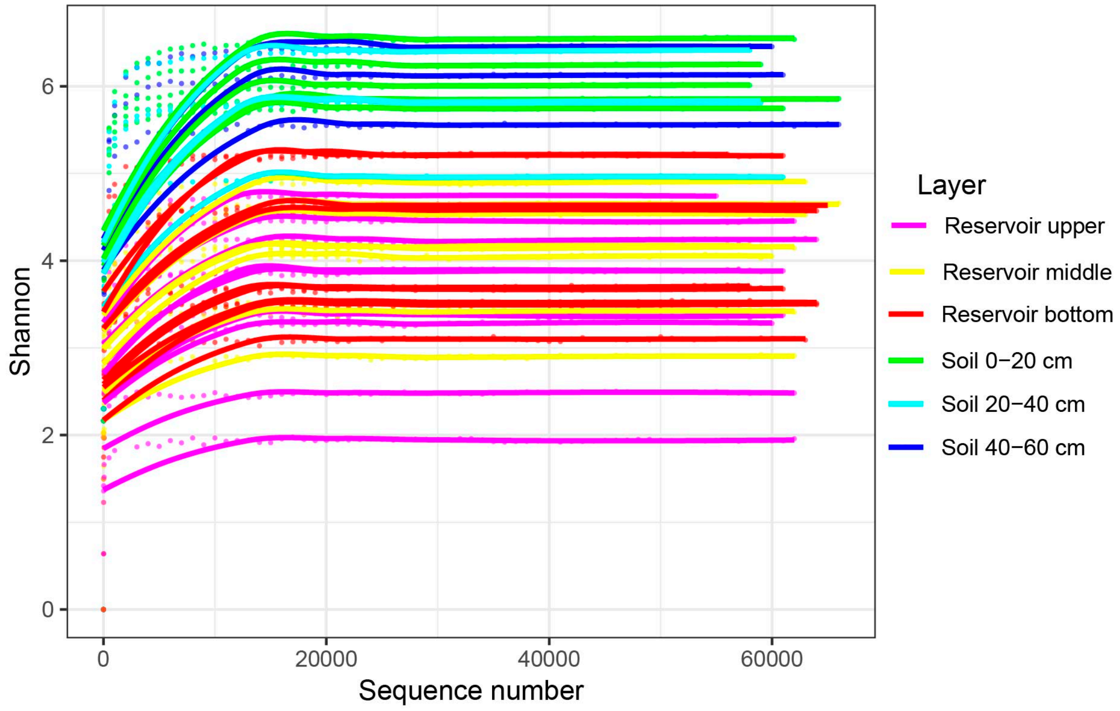

Figure S1. Rarefaction curve of bacterial community Shannon index reaches saturation stage with increasing sequencing depth.

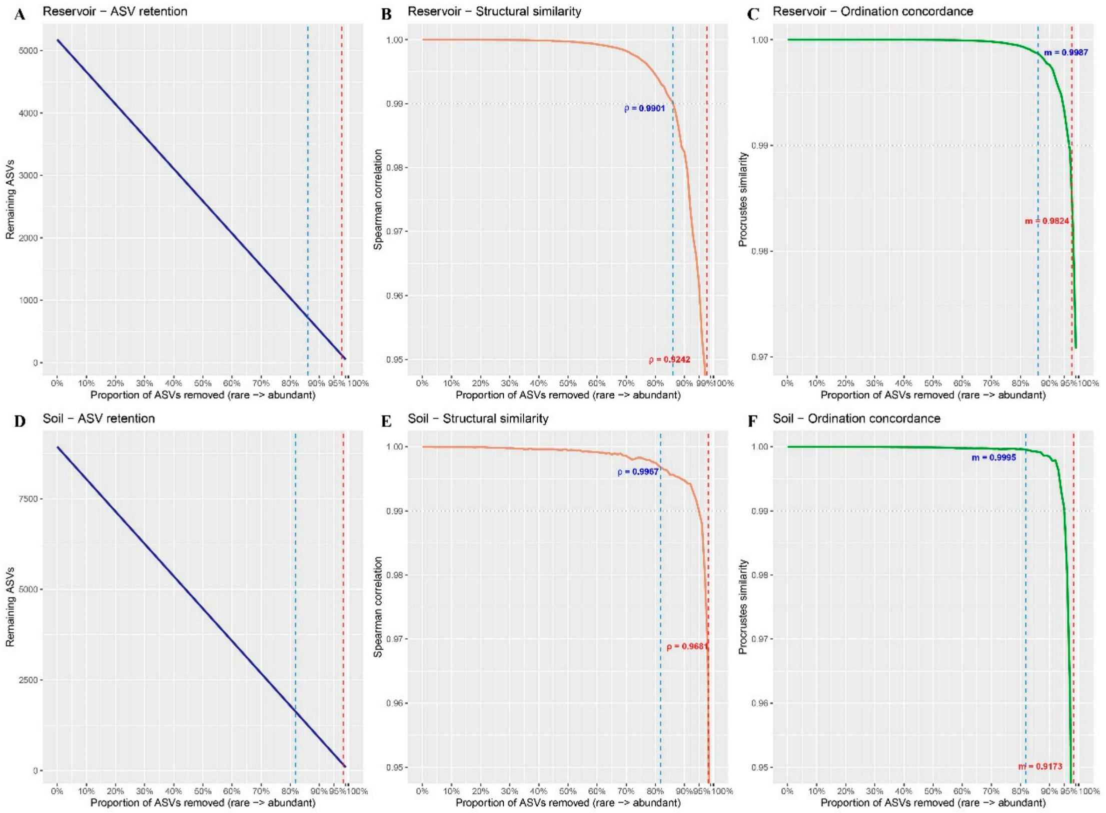

Figure S2. Multivariate cutoff level analysis for the reservoir (A-C) and soil (D-F) communities based on the sequential removal of ASVs from rare to abundant. Panels: (A, D) Number of Remaining ASVs; (B, E) Structural similarity, measured as non-parametric Spearman rank correlations between Bray–Curtis distance matrices of the original and truncated communities; (C, F) Ordination concordance, quantified as Procrustes similarity of principal coordinates. The blue dashed lines indicate the proportion of ASVs classified as rare at a 0.01% mean relative abundance threshold; the blue dashed lines indicate the proportion of ASVs classified as abundant at a 0.1% mean relative abundance threshold in each habitat.

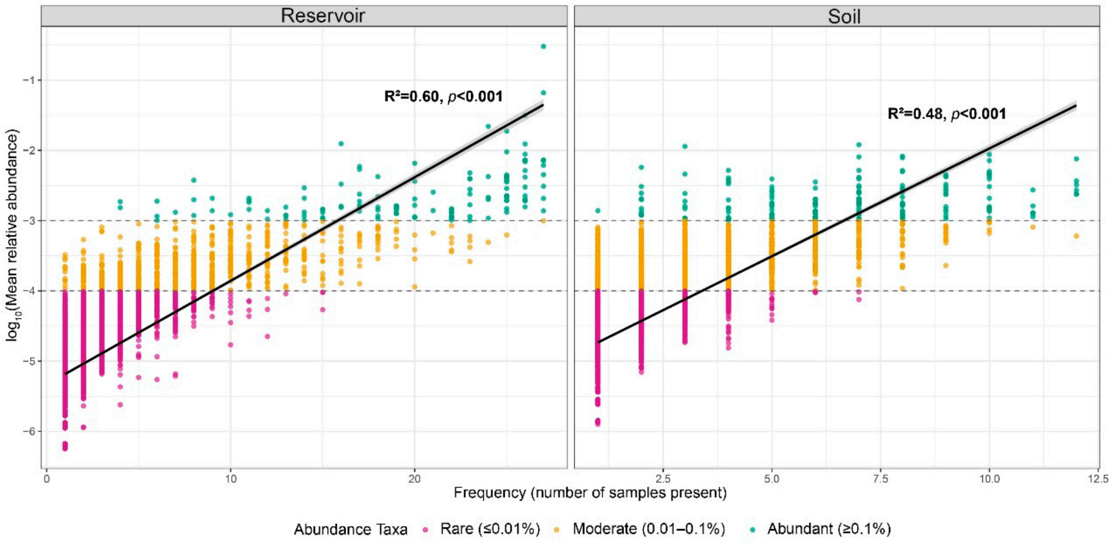

Figure S3. Abundance-Frequency relationships of abundant, moderate, and rare ASVs in reservoir and soil habitats. Each point represents an ASV. The solid black lines show least-squares linear fits on the log10 scale; shaded areas indicate 95% confidence intervals. Horizontal dashed lines mark the abundance thresholds at 0.01% and 0.1%. R^2 is the coefficient of determination for the linear regression, and p denotes the significant level.

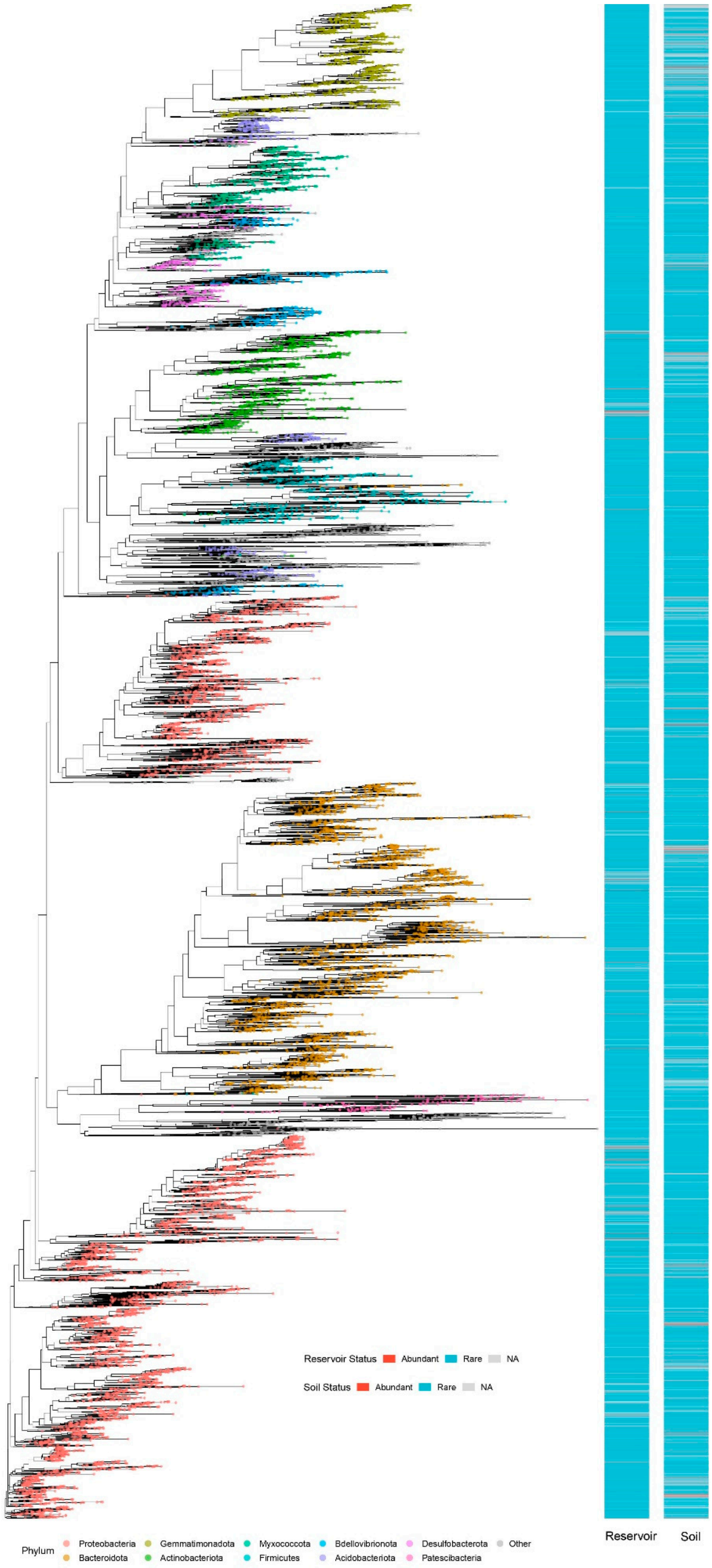

Figure S4. Phylogenetic tree of abundant and rare ASVs in reservoir and soil habitats.

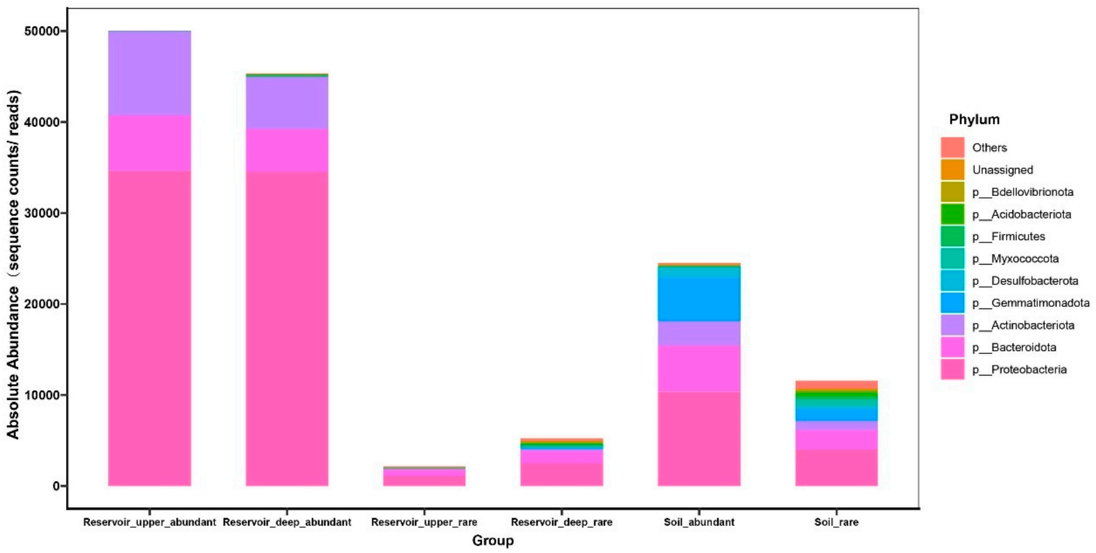

Figure S5. Phylum-level composition of the top 10 abundant and rare bacterial taxa based on sequence counts (reads) across the upper and deep reservoir layers and adjacent soils.

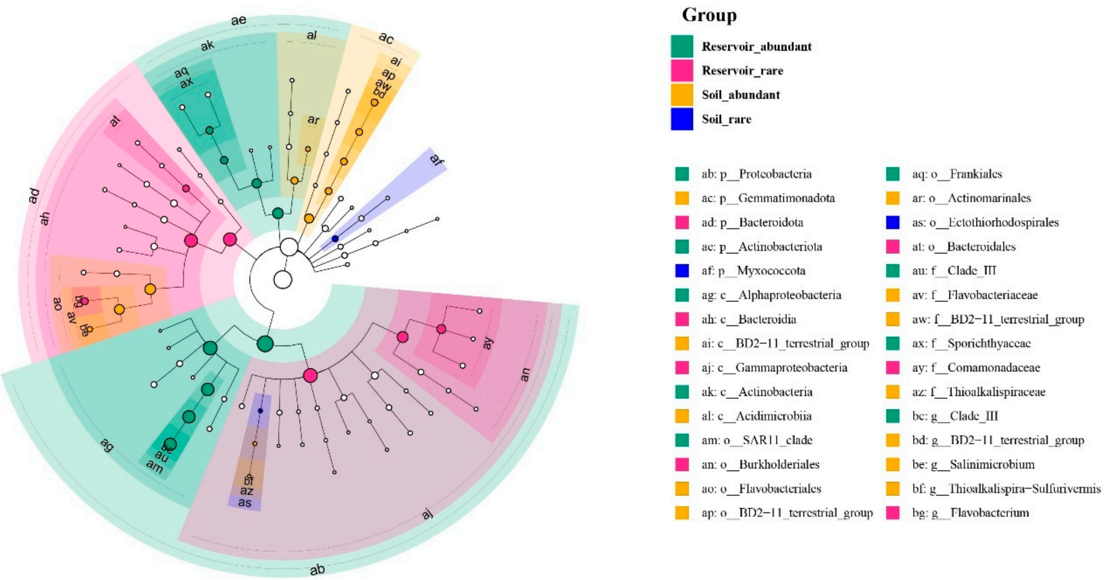

Figure S6. Taxonomic cladogram from Linear discriminant analysis Effect Size (LEfSe) analysis identifying significant biomarker taxa in bacterial communities across four groups: reservoir-abundant, reservoir-rare, soil-abundant, and soil-rare.

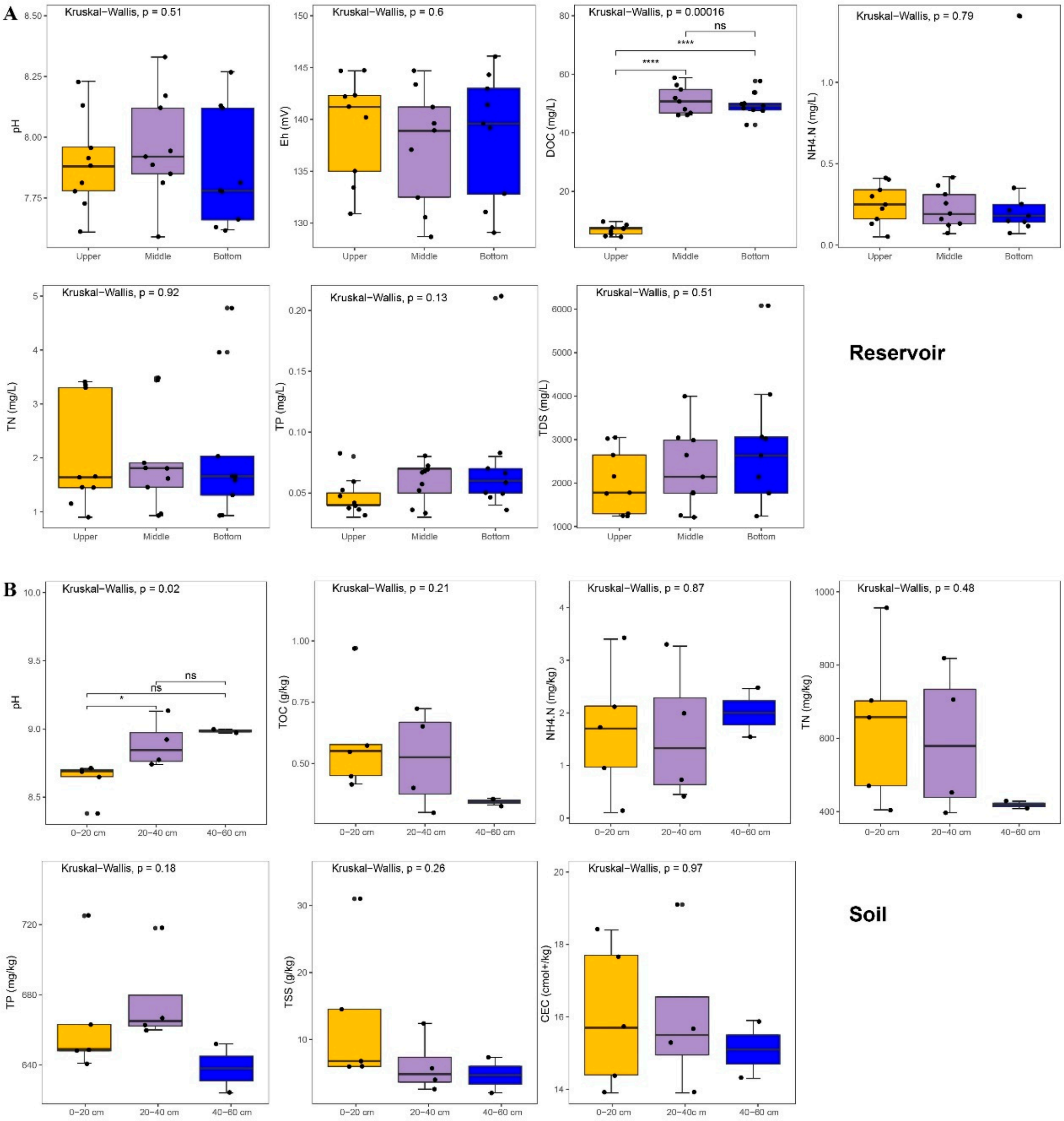

Figure S7. The vertical variation in environmental factors in the reservoir (A) and soil (B). The statistical differences were evaluated among layers utilizing the Kruskal–Wallis test and between layers, utilizing the Wilcoxon rank sum test (ns, not significant, * FDR $p < 0.05$, **** FDR $p < 0.0001$). Eh: oxidation-reduction potential TDS: total dissolved solids; TN: total nitrogen; TP: total phosphorus; DOC: dissolved organic carbon; Eh, oxidation-reduction potential, TSS: total soluble salts, TOC: total organic carbon, CEC: cation exchange capacity.

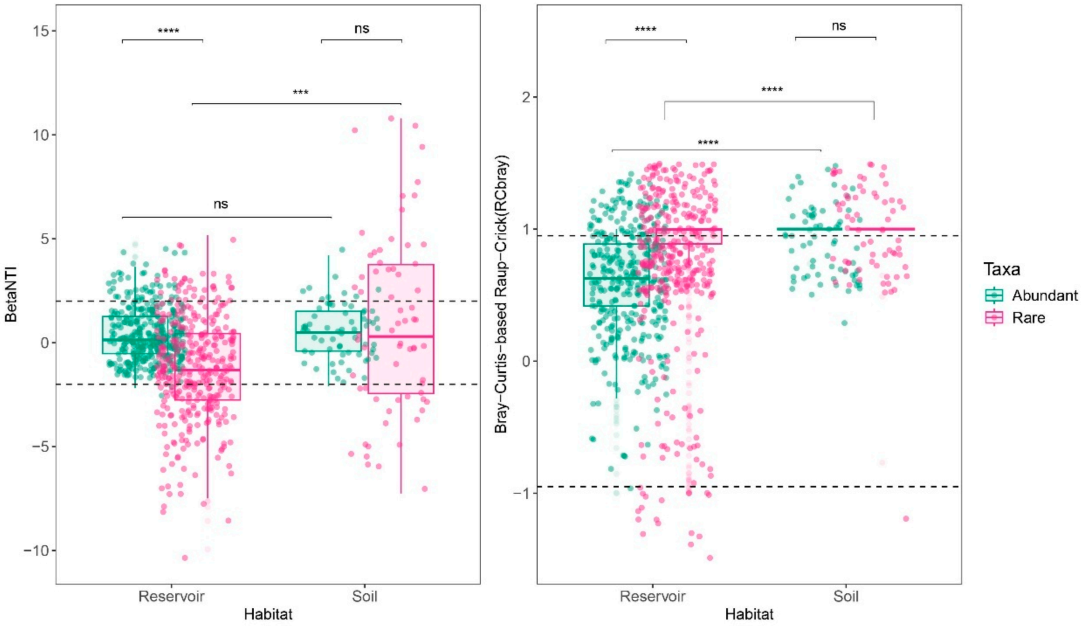

Figure S8. β-nearest taxon index (βNTI) and Bray–Curtis-based Raup–Crick (RCbray) analyses of abundant and rare taxa between reservoir water and soil. Comparisons between abundant and rare taxa, between reservoir and soil with Wilcoxon test (ns: not significant, *** FDR $p < 0.001$, **** FDR $p < 0.0001$).

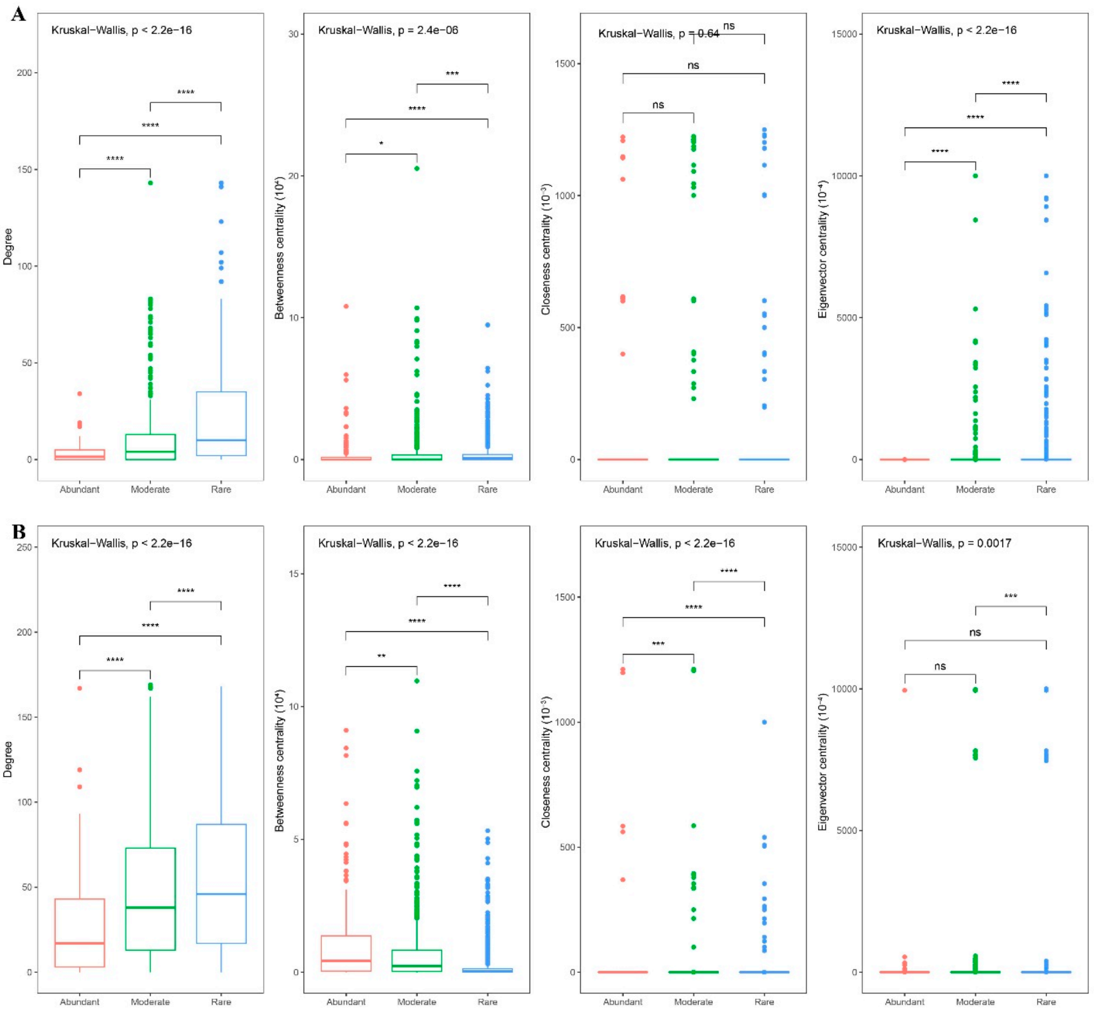

Figure S9. Comparison of non-normalized topological parameters (degree, betweenness centrality, closeness centrality, and eigenvector centrality) among abundant, moderate, and rare taxa in reservoir (A) and soil (B). Statistical differences were assessed using the Kruskal–Wallis's test and Wilcoxon rank sum test (ns: not significant, * FDR $p < 0.05$, ** FDR $p < 0.01$, *** FDR $p < 0.001$, **** FDR $p < 0.0001$).

Supplementary Tables

Table S1 Identification and information of abundant, moderate, and rare ASVs in reservoir and soil habitats. Description of bacterial ASV datasets based on exact sequence variants and frequency of each ASV.

Habitat	Taxa	ASV number (Percentage%)	Sequence number (Percentage%)	Median frequency	Interquartile range (frequency)	Median of mean relative abundance
Reservoir	Abundant	124 (2.40%)	1,266,308 (75.81%)	20.5	10	0.19%
	Moderate	597 (11.54%)	290,317 (17.38%)	8	6	0.02%
	Rare	4454 (86.07%)	113,817 (6.81%)	1	1	0.0007%
Soil	Abundant	162 (1.81%)	294,128 (39.93%)	7	3	0.18%
	Moderate	1459 (16.33%)	303,550 (41.21%)	3	2	0.02%
	Rare	7313 (81.86%)	138,955 (18.86%)	1	0	0.0017%

Note: Abundant taxa were defined as the ASVs with average relative abundances greater than or equal to 0.1% in all samples; Rare taxa were defined as the ASVs with average relative abundances lass than or equal to 0.01% in all samples, the remaining ASVs were designated as moderate taxa.

The values in parentheses indicate the percentage of ASVs number or sequence of different taxa (abundant, moderate, rare) in the total ASVs.

Table S2 Results of PERMANOVA test for effects of habitat (reservoir vs. soil) and taxa abundance types (abundant vs. rare) on bacterial community structure based on Bray–Curtis dissimilarity. Statistical significance: *** $p < 0.001$. Total sample size: 78. Sample sizes: Reservoir Abundant (n=27), Reservoir Rare (n=27), Soil Abundant (n=12), Soil Rare (n=12).

Term	Df	Sum of squares	R^2	F	*p* value
Habitat	1	2.890	0.084	8.919	0.001***
Taxa	1	4.599	0.134	14.191	0.001***
Habitat*Taxa	1	2.911	0.085	8.982	0.001***
Residual	74	23.981	0.698		
Total	77	34.81			

Table S3 Pairwise PERMANOVA comparisons of bacterial community structure among groups defined by habitat (reservoir, soil) and taxa abundance type (abundant, rare), based on the Bray–Curtis dissimilarity. The p-values were adjusted for multiple comparisons using the Benjamini–Hochberg false discovery rate (FDR) method. Statistical significance levels: ns, not significant; * $p < 0.05$.

Group comparison	F	R^2	*p*. adjusted	Significance
Reservoir Abundant vs Reservoir Rare	0.471	0.009	1.000	ns
Reservoir Abundant vs Soil Abundant	3.512	0.087	0.003	*
Reservoir Abundant vs Soil Rare	3.509	0.087	0.003	*
Reservoir Rare vs Soil Abundant	3.417	0.085	0.003	*
Reservoir Rare vs Soil Rare	3.456	0.085	0.003	*
Soil Abundant vs Soil Rare	0.747	0.033	1.000	ns

Sample sizes (used for all pairwise tests): Reservoir Abundant (n=27), Reservoir Rare (n=27), Soil Abundant (n=12), Soil Rare (n=12).

Table S4 Dissimilarity test of abundant and rare taxa in the reservoir water by ANOISM (analysis of similarity), and PERMANOVA (permutational multivariate analysis of variance) based on Bray–Curtis dissimilarity matrix between different water layers. Each water layer includes 9 samples for abundant taxa and 9 samples for rare taxa. Significance codes reflect adjusted *p*-values: *FDR $p < 0.05$; **FDR $p < 0.01$.

**Taxa**	**Water layer**	**ANOISM**		**PERMANOVA**	
		r	*p*	F	*p*
Abundant	Upper *vs*. Middle	0.293	0.003**	3.851	0.015*
	Upper *vs*. Bottom	0.255	0.008**	3.515	0.009**
	Middle *vs*. Bottom	-0.099	1.000	0.226	0.986
Rare	Upper *vs*. Middle	0.765	0.001***	2.127	0.002**
	Upper *vs*. Bottom	0.840	0.001***	2.071	0.002**
	Middle *vs*. Bottom	-0.052	0.733	0.740	0.955

Table S5 Taxonomic information of shared ASVs between reservoir_abundant and soil_abundant, reservoir_abundant and soil_rare, reservoir_rare and soil_abundant.

ASV_ID	Phylum	Class	Order	Family	Genus
**Shared between Reservoir_abundant and_Soil_abundant**					
ASV5	Proteobacteria	Gammaproteobacteria	Burkholderiales	Burkholderiaceae	Limnobacter
**Shared between Reservoir_rare and Soil_abundant**					
ASV32	Proteobacteria	Gammaproteobacteria	Pseudomonadales	Pseudomonadaceae	Thioalkalispira-Sulfurivermis
ASV39	Proteobacteria	Gammaproteobacteria	Ectothiorhodospirales	Thioalkalispiraceae	Pelagibius
ASV179	Proteobacteria	Alphaproteobacteria	Kiloniellales	Kiloniellaceae	Salinimicrobium
ASV65	Bacteroidota	Bacteroidia	Flavobacteriales	Flavobacteriaceae	Alcanivorax
ASV35	Proteobacteria	Gammaproteobacteria	Oceanospirillales	Alcanivoracaceae1	uncultured
ASV91	Desulfobacterota	Desulfobulbia	Desulfobulbales	Desulfobulbaceae	uncultured
ASV96	Proteobacteria	Alphaproteobacteria	uncultured	uncultured	uncultured
ASV353	Actinobacteriota	Acidimicrobiia	Actinomarinales	uncultured	
**Shared between Reservoir_abundant and Soil_rare**					
ASV3	Actinobacteriota	Actinobacteria	Frankiales	Sporichthyaceae	Perlucidibaca
ASV6	Proteobacteria	Gammaproteobacteria	Pseudomonadales	Moraxellaceae	CL500-29_marine_group
ASV12	Actinobacteriota	Acidimicrobiia	Microtrichales	Ilumatobacteraceae	hgcI_clade
ASV15	Actinobacteriota	Actinobacteria	Frankiales	Sporichthyaceae	Limnohabitans
ASV17	Proteobacteria	Gammaproteobacteria	Burkholderiales	Comamonadaceae	hgcI_clade
ASV23	Actinobacteriota	Actinobacteria	Frankiales	Sporichthyaceae	Aquabacterium
ASV30	Proteobacteria	Gammaproteobacteria	Burkholderiales	Comamonadaceae	Sphingorhabdus
ASV40	Proteobacteria	Alphaproteobacteria	Sphingomonadales	Sphingomonadaceae	Algoriphagus
ASV53	Bacteroidota	Bacteroidia	Cytophagales	Cyclobacteriaceae	Candidatus_Aquirestis

ASV54	Bacteroidota	Bacteroidia	Chitinophagales	Saprospiraceae	Candidatus_Nitrotoga
ASV58	Proteobacteria	Gammaproteobacteria	Burkholderiales	Gallionellaceae	hgcI_clade
ASV63	Actinobacteriota	Actinobacteria	Frankiales	Sporichthyaceae	Hydrogenophaga
ASV75	Proteobacteria	Gammaproteobacteria	Burkholderiales	Comamonadaceae	Flavobacterium
ASV66	Bacteroidota	Bacteroidia	Flavobacteriales	Flavobacteriaceae	GKS98_freshwater_group
ASV76	Proteobacteria	Gammaproteobacteria	Burkholderiales	Alcaligenaceae	Flavobacterium
ASV89	Bacteroidota	Bacteroidia	Flavobacteriales	Flavobacteriaceae	Polaromonas
ASV100	Proteobacteria	Gammaproteobacteria	Burkholderiales	Comamonadaceae	Paraperlucidibaca
ASV11	Proteobacteria	Gammaproteobacteria	Pseudomonadales	Moraxellaceae	Reyranella
ASV115	Proteobacteria	Alphaproteobacteria	Reyranellales	Reyranellaceae	Flavobacterium
ASV119	Bacteroidota	Bacteroidia	Flavobacteriales	Flavobacteriaceae	Hydrogenophaga
ASV117	Proteobacteria	Gammaproteobacteria	Burkholderiales	Comamonadaceae	
ASV122	Proteobacteria	Alphaproteobacteria	Rhodobacterales	Rhodobacteraceae	uncultured
ASV164	Actinobacteriota	Acidimicrobiia	Microtrichales	uncultured	uncultured
ASV171	Bacteroidota	Bacteroidia	Cytophagales	Cyclobacteriaceae	Nevskia
ASV191	Proteobacteria	Gammaproteobacteria	Salinisphaerales	Solimonadaceae	Nitrosomonas
ASV209	Proteobacteria	Gammaproteobacteria	Burkholderiales	Nitrosomonadaceae	Flavobacterium
ASV222	Bacteroidota	Bacteroidia	Flavobacteriales	Flavobacteriaceae	Polaribacter
ASV218	Bacteroidota	Bacteroidia	Flavobacteriales	Flavobacteriaceae	Hyphomonas
ASV134	Proteobacteria	Alphaproteobacteria	Caulobacterales	Hyphomonadaceae	Nitrosomonas
ASV203	Proteobacteria	Gammaproteobacteria	Burkholderiales	Nitrosomonadaceae	GKS98_freshwater_group
ASV247	Proteobacteria	Gammaproteobacteria	Burkholderiales	Alcaligenaceae	

Table S6 Environmental factors of three water layers (upper, middle, and bottom) in 27 samples, of three layers (0–20 cm, 20–40 cm, and 40–60 cm) of adjacent soil in 11samples, presented as mean ± standard deviation. Eh: oxidation-reduction potential, TDS: total dissolved solids; TN: total nitrogen; TP: total phosphorus; DOC: dissolved organic carbon; Eh, oxidation-reduction potential, TSS: total soluble salts, TOC: total organic carbon, CEC: cation exchange capacity.

Habitat	Layer	pH	Eh (mV)	DOC (mg/L)	NH_4^+–N (mg/L)	TN (mg/L)	TP (mg/L)	TDS (mg/L)
Reservoir	Upper	7.89±0.18	139.40±4.76	6.82±1.64	0.25±0.12	2.04±0.96	0.05±0.01	2021.51±735.40
	Middle	7.96±0.21	137.41±5.34	51.05±4.48	0.23±0.12	1.94±0.88	0.06±0.02	2314.30±926.30
	Bottom	7.87±0.23	138.51±5.75	49.67±3.92	0.32±0.39	2.10±1.28	0.08+0.05	2861.22±1474.91
Habitat	Layer	pH	TOC (g/kg)	NH_4^+–N (mg/kg)	TN (mg/kg)	TP (mg/kg)	TSS (g/kg)	CEC (cmol+/kg)
Soil	0—20 cm	8.63±0.14	0.59±0.22	1.66±1.24	638±216.68	665±34.37	12.84±10.77	16.02±1.98
	20—40 cm	8.89±0.18	0.52±0.20	1.60±1.30	593±201.40	677±27.48	6.17±4.35	16.00±2.21
	40—60 cm	8.99±0.02	0.34±0.02	2.00±0.65	418±14.14	638±19.80	4.68±3.75	15.10±1.13

Table S7 Comparison of topological properties for the empirical co-occurrence networks of bacterioplankton communities in the reservoir water and soil and their associated random networks.

	Parameters	Reservoir	Soil
**Empirical network**	Nodes	2143	2561
	Abundant%	5.79%	6.29%
	Moderate%	27.30%	46.66%
	Rare%	66.92%	47.05%
	Edges	20,218	64,757
	Positive edges	20,195	64,149
	Negative edges	23	608
	Average degree	18.87	50.57
	Average weight degree	16.79	46.70
	Average path length	5.95	4.24
	Network diameter	15.38	13.59
	Network density	0.01	0.02
	Clustering coefficient	0.70	0.86
	Betweenness centralization	0.08	0.04
	Centralization degree	0.06	0.05
	Network modularity	0.69	0.74
**Random network**	Clustering coefficient	0.0088	0.0198
		(SD = 0.0003)	(SD=0.0001)
	Average path length	2.889	2.341
		(SD = 0.0005)	(SD=0.0002)
	Modularity	0.1457	0.0723
		(SD = 0.0028)	(SD= 0.0011)
	Network diameter	4.041	3.00
		(SD=0 .1985)	(SD=0.000)
	Network density	0.0088	0.00198
		(SD = 0.0000)	(SD= 0.000)

Note: Number of ASVs with the correlation |r| > 0.8 and statistical significance (p< 0.05); In random network, the numbers in parentheses represent the standard deviation.

Table S8 Taxonomic information of keystone taxa from the networks in the reservoir water and soil.

ASV_ID	Taxa	Node roles	Phylum	Class	Order	Family	Genus
**Reservoir water**							
ASV163	Abundant	Module hubs	Proteobacteria	Gammaproteobacteria	Burkholderiales	Burkholderiaceae	Polynucleobacter
ASV187	Abundant	Module hubs	Proteobacteria	Alphaproteobacteria	Rhizobiales	Rhizobiales_Incertae_Sedis	uncultured
ASV219	Abundant	Connectors	Proteobacteria	Gammaproteobacteria	Xanthomonadales	Xanthomonadaceae	Arenimonas
ASV909	Moderate	Module hubs	Proteobacteria	Alphaproteobacteria	Acetobacterales	Acetobacteraceae	Rhodovastum
ASV1729	Moderate	Connectors	Proteobacteria	Gammaproteobacteria	Burkholderiales	TRA3-20	TRA3-20
ASV1825	Moderate	Connectors	Bacteroidota	Bacteroidia	Chitinophagales	uncultured	uncultured
ASV2525	Rare	Connectors	Bacteroidota	Bacteroidia	Cytophagales	Microscillaceae	OLB12
ASV2933	Rare	Connectors	Actinobacteriota	Actinobacteria	Propionibacteriales	Nocardioidaceae	Aeromicrobium
ASV3158	Rare	Module hubs	Proteobacteria	Gammaproteobacteria	Burkholderiales	Rhodocyclaceae	Sulfuritalea
ASV3718	Rare	Module hubs	Proteobacteria	Alphaproteobacteria	Rhodobacterales	Rhodobacteraceae	Tabrizicola
ASV3873	Rare	Connectors	Gemmatimonadota	Gemmatimonadetes	Gemmatimonadales	Gemmatimonadaceae	uncultured
ASV2130	Rare	Connectors	Acidobacteriota	Acidobacteriae	PAUC26f	PAUC26f	PAUC26f
ASV4065	Rare	Connectors	Bacteroidota	Bacteroidia	Cytophagales	Microscillaceae	uncultured
ASV4068	Rare	Connectors	Proteobacteria	Alphaproteobacteria	uncultured	uncultured	uncultured
ASV4199	Rare	Module hubs	Proteobacteria	Gammaproteobacteria	Burkholderiales	Rhodocyclaceae	ssigned
ASV4372	Rare	Module hubs	Proteobacteria	Gammaproteobacteria	Xanthomonadales	Rhodanobacteraceae	Ahniella
ASV4586	Rare	Connectors	Proteobacteria	Alphaproteobacteria	Rhodobacterales	Rhodobacteraceae	ssigned
ASV6228	Rare	Module hubs	Myxococcota	Myxococcia	Myxococcales	Myxococcaceae	P3OB-42
ASV7266	Rare	Connectors	Gemmatimonadota	BD2-11_terrestrial_group	BD2-11_terrestrial_group	BD2-11_terrestrial_group	BD2-11_terrestrial_group
ASV7424	Rare	Module hubs	NB1-j	NB1-j	NB1-j	NB1-j	NB1-j

ASV7451	Rare	Connectors	Proteobacteria	Alphaproteobacteria	Rhizobiales	Xanthobacteraceae	Bradyrhizobium
ASV8705	Rare	Connectors	Bdellovibrionota	Bdellovibrionia	Bdellovibrionales	Bdellovibrionaceae	Bdellovibrio
ASV7623	Rare	Connectors	Proteobacteria	Gammaproteobacteria	Enterobacterales	Yersiniaceae	Serratia
**Soil**							
ASV436	Abundant	Connectors	Bacteroidota	Bacteroidia	Flavobacteriales	Flavobacteriaceae	Gramella
ASV3620	Rare	Connectors	Proteobacteria	Alphaproteobacteria	Kiloniellales	Kiloniellaceae	uncultured
ASV3770	Rare	Connectors	NB1-j	NB1-j	NB1-j	NB1-j	NB1-j
ASV4026	Rare	Connectors	Latescibacterota	Latescibacterota	Latescibacterota	Latescibacterota	Latescibacterota
ASV4434	Rare	Connectors	Gemmatimonadota	BD2-11_terrestrial_group	BD2-11_terrestrial_group	BD2-11_terrestrial_group	BD2-11_terrestrial_group
ASV7682	Rare	Connectors	Acidobacteriota	Acidobacteriae	Bryobacterales	Bryobacteraceae	Bryobacter
ASV9355	Rare	Connectors	Proteobacteria	Gammaproteobacteria	EPR3968-O8a-Bc78	EPR3968-O8a-Bc78	EPR3968-O8a-Bc78
ASV571	Moderate	Connectors	Bacteroidota	Rhodothermia	Balneolales	Balneolaceae	Gracilimonas
ASV595	Moderate	Connectors	Proteobacteria	Gammaproteobacteria	Nitrosococcales	Nitrosococcaceae	ssigned
ASV860	Moderate	Connectors	Myxococcota	bacteriap25	bacteriap25	bacteriap25	bacteriap25
ASV970	Moderate	Connectors	Gemmatimonadota	BD2-11_terrestrial_group	BD2-11_terrestrial_group	BD2-11_terrestrial_group	BD2-11_terrestrial_group
ASV762	Moderate	Connectors	Bacteroidota	Bacteroidia	Chitinophagales	Chitinophagaceae	Sediminibacterium
ASV1091	Moderate	Connectors	Proteobacteria	Alphaproteobacteria	Defluviicoccales	uncultured	uncultured
ASV854	Moderate	Connectors	Patescibacteria	Parcubacteria	ssigned	ssigned	ssigned
ASV1220	Moderate	Connectors	Bacteroidota	Bacteroidia	Flavobacteriales	Flavobacteriaceae	Salinimicrobium
ASV1347	Moderate	Connectors	Actinobacteriota	Acidimicrobiia	Actinomarinales	uncultured	uncultured
ASV1390	Moderate	Connectors	Bacteroidota	Rhodothermia	Rhodothermales	Rhodothermaceae	uncultured
ASV1397	Moderate	Connectors	Bacteroidota	Bacteroidia	Sphingobacteriales	NS11-12_marine_group	NS11-12_marine_group
ASV1889	Moderate	Connectors	Proteobacteria	Gammaproteobacteria	Ectothiorhodospirales	Thioalkalispiraceae	Thioalkalispira-Sulfurivermis
ASV2367	Moderate	Connectors	Bacteroidota	Rhodothermia	Balneolales	Balneolaceae	uncultured
ASV2207	Moderate	Connectors	Proteobacteria	Alphaproteobacteria	Sphingomonadales	Sphingomonadaceae	Altererythrobacter

ASV2654	Moderate	Connectors	Bacteroidota	Bacteroidia	Chitinophagales	uncultured	uncultured
ASV2683	Moderate	Connectors	Proteobacteria	Alphaproteobacteria	ssigned	ssigned	ssigned
ASV2686	Moderate	Connectors	Bacteroidota	Bacteroidia	Flavobacteriales	Cryomorphaceae	Vicingus
ASV2922	Moderate	Module hubs	Gemmatimonadota	AKAU4049	AKAU4049	AKAU4049	AKAU4049
ASV2996	Moderate	Connectors	Desulfobacterota	Desulfuromonadia	Bradymonadales	Bradymonadales	Bradymonadales

Table S9 Node level topology characteristics of abundant, moderate, and rare taxa in the bacterial networks of reservoir and soil.

Node level topology characteristics	Reservoir			Soil		
	Abundant	Moderate	Rare	Abundant	Moderate	Rare
Nodes	124	585	1434	161	1195	1205
Edges related to taxa	352	5276	18787	4520	44819	47770
Edge/node ratio	2.839	9.019	13.101	28.075	37.505	39.643
Average degree	3.419	10.942	23.439	28.143	48.799	55.326
Average between centrality	4551.597	4948.568	3423.583	11643.985	6221.679	2408.107
Average closeness centrality	0.148	0.065	0.043	0.034	0.007	0.008
Average eigenvector centrality	0.000	0.015	0.071	0.007	0.061	0.073
Average clustering coefficient	0.210	0.321	0.525	0.397	0.636	0.850
